# Fighting Fire with Fire: Impact of Sugary Diets on Metabolically Deranged Mice

**DOI:** 10.3390/nu17010100

**Published:** 2024-12-30

**Authors:** John I. Glendinning, Niki Williams

**Affiliations:** 1 Department of Biology, Barnard College, Columbia University, New York, NY 10027, USA; 2 Department of Neuroscience & Behavior, Barnard College, Columbia University, New York, NY 10027, USA; nikiwilliams5@gmail.com

**Keywords:** glucose tolerance, insulin sensitivity, hyperinsulinemia, cellulosic vs. corn sugars, sugar avidity

## Abstract

**Background/Objectives:** There is controversy about the health risks of sugary diets. A recent study reported that chronic consumption of 11% sugar solutions improved glycemic control in lean mice. Based on this finding, we hypothesized that chronic consumption of the same 11% sugar solutions would also improve glycemic control in metabolically deranged mice. **Methods:** We exposed mice to a high-fat/high-sugar diet for 12 weeks. Then, we switched the mice to a control (i.e., standard chow) or one of four experimental diets for 8 weeks. The experimental diets contained standard chow plus an 11% solution of glucose or high-fructose syrup. The sugar syrups were derived from corn or cellulose. We included the cellulosic syrups because they contain polyphenols, which are thought to promote glycemic control. We measured body weight, adiposity, glucose tolerance, insulinemia, insulin sensitivity, body composition, and avidity for sweeteners. **Results:** Mice switched to the control diet lost weight, whereas mice switched to the experimental diets remained obese and hyperinsulinemic. Thus, the experimental diets did not cause the mice to regain normal metabolic health. Nevertheless, we observed (i) improvements in glucose tolerance in mice on both the control and experimental diets; (ii) reduced insulinemia and enhanced insulin sensitivity in mice offered the cellulosic syrups; (iii) elevations in cephalic-phase insulin responses in mice on the experimental diets; and (iv) increased avidity for sweeteners in mice on the control but not the experimental diets. **Conclusions:** Switching metabolically deranged mice to the experimental diets, particularly those with cellulosic sugars, improved glucose tolerance and insulin sensitivity.

## 1. Introduction

Foods high in both sugar and fat are thought to cause overconsumption, adiposity, and insulin intolerance ([1,2,3,4,5,6]; but see [7,8]). They also diminish olfactory function [9] and avidity for sugars [10] in rodents when present chronically in the diet. The health risks of foods high in sugar alone are less clear [2,11,12]. In one mouse study, high-sugar diets caused less weight gain and adiposity than did isocaloric diets high in sugar and fat [13]. There are also contradictory reports about the health risks of some commonly consumed sugar syrups. For example, despite the widespread belief that high-fructose corn syrups are more likely to cause obesity and insulin resistance than other types of sugar [14,15,16,17], support for this claim is equivocal [11,12,18,19,20]. The sugar debate is further complicated by the diversity of sugars in the modern diet. In addition to mono- and disaccharides (e.g., glucose, fructose, lactose, and sucrose), there are sugar alcohols (e.g., sorbitol and mannitol), rare sugars (e.g., allulose and isomaltulose), and various syrups (e.g., honey, maple, agave, glucose and high-fructose). All of these sugars have different metabolic fates in the body [21,22].

Glucose (Gluc) and high-fructose (HiFruc) syrups are added to many foods and beverages [22]. These syrups are typically derived from corn [23], but they can also be derived from recycled biomass (i.e., the cellulose, hemicellulose, and lignin in agricultural waste). The conversion of recycled biomass into so-called “cellulosic” syrups is a new technology [24]. It is of interest because cellulosic syrups represent a sustainable resource and contain several minor components, including polyphenols (i.e., condensed and hydrolysable tannins) [25]. Dietary polyphenols are thought to provide protection against diet-induced obesity, insulin resistance, and hepatic steatosis [26,27,28,29,30]. Further, when polyphenols were added to a high-fructose chow, hyperglycemic rats experienced improvements in fasting blood glucose levels, body weight, and adiposity [31]. What remains unclear, however, is whether the polyphenols in cellulosic syrups produce similar health benefits.

The present study was motivated by two prior studies, which reported that chronically consuming 11% solutions of some but not all sugars improved blood glucose control [20] and reduced avidity for sugars [32]. In these studies, lean C57BL/6 (B6) mice were offered a Gluc syrup derived from cellulose (Gluc-Cell) or corn (Gluc-Corn), or a HiFruc syrup derived from cellulose (HiFruc-Cell) or corn (HiFruc-Corn). See Table 1 for the composition of these syrups. Consumption of the Gluc-Cell and Gluc-Corn syrups enhanced glucose tolerance, cephalic-phase insulin responses (CPIRs), insulin sensitivity, and adiposity, whereas consumption of the HiFruc-Cell (but not the HiFruc-Corn) syrups enhanced blood glucose control [20]. Consumption of all four sugar syrups also reduced avidity for sweet solutions, as indicated by lower lick rates and numbers of trials initiated during brief-access lick tests [32]. Despite the diet-induced reductions in avidity for the sweet solutions, the mice increased daily consumption of the Gluc and HiFruc syrups across the experiment [32].

Four important observations emerge from the foregoing discussion. (i) Diets high in sugar + fat appear to promote obesity, glucose intolerance and hyperinsulinemia more effectively than isocaloric diets high in sugar alone. (ii) Consumption of some sugar syrups can improve glycemic control in lean mice, in part by increasing CPIR magnitude and insulin sensitivity. (iii) Diets containing polyphenols can lower fasting blood glucose levels in rodents with glucose intolerance. (iv) Chronic consumption of sugar solutions reduces avidity for sweet tasting solutions, but not daily intake of sugar solutions.

Based on these observations, we hypothesized that chronic consumption of diets supplemented with 11% sugar solutions would enhance glycemic control, CPIR magnitudes, and insulin sensitivity in metabolically deranged mice. To test this hypothesis, we induced metabolic derangement by exposing the mice to a high-fat/high-sugar (HF/HS) diet for 12 weeks. Then, we switched the mice to a control diet (i.e., standard chow) or one of four experimental diets for 8 weeks. The experimental diets contained standard chow plus an 11% solution of Gluc-Corn, Gluc-Cell, HiFruc-Corn, or HiFruc-Cell syrup.

## 2. Materials and Methods

### 2.1. Animals and Housing Conditions

We tested a total of 24 male and 24 female mice from the C57Bl/6 (B6) inbred strain. The mice were derived from stock purchased from Jackson Laboratories (Bar Harbor, ME, USA). Each treatment level contained an equal number of both sexes. All mice were naïve to pure sugars and sweeteners prior to testing. For all analyses, the experimental unit was a single mouse. An *a priori* power analysis revealed that we needed 8 mice per treatment group to detect an effect of diet on the glucose tolerance.

The mice were kept in a vivarium with controlled temperature and humidity on a 12:12 h light-dark cycle (lights on at 6 a.m.). Mice were housed individually in polycarbonate cages (27.5 × 17 × 12.5 cm) with Bed-O’Cobs bedding (Andersons, Maumee, OH, USA; andersonslabbedding.com) and cotton pads (Ancare, Bellmore, NY, USA; www.ancare.com). The mice had unlimited access to tap water and standard chow (5001, PMI Nutrition International, St. Louis, MO, USA; www.labdiet.com), unless noted otherwise. The standard chow had an energy density of 3.36 kcal/g and the following macronutrient composition (as a % of total mass): sugars (6.2%), corn starch (31.9%), fat (5%), and protein (23.9%). The % of kcal from each macronutrient was carbohydrate (58.0%), fat (13.5%), and protein (28.5%). Mice obtained water from sipper spouts (with a 1.5 mm hole) attached to water bottles placed on the cage-top.

The HF/HS chow (Teklad diet: TD.08811; Madison, WI, USA; www.envigo.com) had an energy density of 4.7 kcal/g. It contained the following macronutrient composition (as a % of total mass): sucrose (34%), corn starch and maltodextrin (11.7%), fat (23%), and protein (20%). The % kcal from each macronutrient was carbohydrate (44.6%), fat (40.7%), and protein (14.7%). Prior work reported that rodents not only increased daily caloric intake on this diet [33], but also developed higher body weight, adiposity, hyperglycemia, and hyperinsulinemia [34].

### 2.2. Sugar Solutions

The Gluc-Corn, Gluc-Cell, HiFruc-Corn, and HiFruc-Cell syrups were diluted to an 11% sugar concentration. This concentration is similar to that found in many commercial beverages [35,36]. We presented the sugar syrups in water, rather than dry diet, because they promote overconsumption and weight gain more effectively in water [37,38]. All the solutions were tested at room temperature. When converting the sugar solution intake (in g) to kcal units, we assumed that the sugars contained a caloric density of 4 kcal/g.

When making the sugar solutions, we dissolved the syrups in deionized water. The glucose syrups contained 95–99% glucose, while the high-fructose syrups contained 40–45% glucose and 55% fructose (see Table 1). The manufacturer of the two cellulosic syrups (Comet, Schaumburg, IL, USA; comet-bio.com) determined that they contained approximately 84.3 μM gallic acid, 93.0 μM protocatechuic acid, and 103.8 μM 4-hydroxybenzoic acid. These polyphenols have been reported to reduce blood sugar levels in rats [31,39,40].

### 2.3. Oral Glucose Tolerance Test (GTT)

During the oral GTT, each mouse took 200 licks from a 1M glucose solution. Given that B6 mice obtain an average of 1.1 μL/lick, it follows that they obtained 0.22 mL of 1M glucose from the 200 licks. This results in a dose of ~40 mg of glucose per 35 g mouse (or 1.14 mg/g). We used this method of oral administration because it mimics the standard approach in human studies, and because it produces better glucose tolerance than IG administration in mice [41]. While this administration method did not control for individual differences in body weight, we ensured that there were no systematic differences in the body weights of mice across the treatment groups at the start of Experiment 1B.

The mice were water-deprived 23 h prior to the GTT so as to motivate licking for the 1M glucose solution (see below). In addition, the mice were food-deprived 6 h prior to the GTT to minimize food in the gastrointestinal tract at the time of testing. All GTTs were conducted between 1:00 and 4:00 p.m.

To ensure that mice licked reliably for 1M glucose during the GTT, we trained them to lick in a gustometer (Mouse Davis Rig; Med-Associates, Inc., Fairfax, VT, USA; www.med-associates.com). To this end, we subjected each mouse to 3 training sessions on separate days. Each one began when the mouse took its first lick, and lasted 30 min. Throughout each training session, the mouse could obtain water from a single sipper tube. The mouse was water-deprived for 22.5 h prior to each training session to motivate licking. Following each session, the mouse was returned to its home cage and given 1 h of access to water and food; it was subsequently water-deprived for 22.5 h. Once training was complete, the mouse was given at least one recovery day.

Immediately prior to the GTT, we measured baseline blood glucose levels and collected blood samples for the plasma insulin assay. Subsequently, we put the mouse in the gustometer and permitted it to take 200 licks from the 1M glucose solution. The mice typically completed the 200 licks within 2 min, but in some cases (<15% of total) they took up to 5 min to do so. Next, the mouse was returned to its home cage without chow or water. At four subsequent time-points (i.e., 5, 15, 30, and 60 min after the mouse initiated licking), we measured blood glucose and collected blood samples for the insulin assay. After the GTT, each mouse received chow and water.

### 2.4. Measuring Blood Glucose and Insulin Levels

To measure blood glucose, a single drop of tail blood was applied to a hand-held glucometer (OneTouch Ultra, Milpitas, CA, USA; www.onetouch.com). This glucometer provides blood glucose readings that closely match those provided by a laboratory biochemical test [42].

For the ultra-sensitive mouse insulin ELISA (Crystal Chem, Elk Grove Village, IL, USA; crystalchem.com), we needed 30 µL of blood for each time-point of the GTT. To this end, we collected tail blood samples in EDTA-coated capillary tubes (Innovative Medical Technologies, Inc., Omaha, NE, USA; innovativemedtech.com). The blood samples were initially stored in ice. After centrifugation (6 min at 6000 rpm), the plasma was stored at −80 °C.

We inferred that a stimulus elicited a CPIR if it induced a significant increase in insulin concentration between the 0 and 5 min time-points of the GTT. We selected the 5 min duration based on the observations that (i) elevations in blood glucose within 5 min of initiating licking for 1M glucose are not sufficient to elicit insulin release within the same time period [43,44], and (ii) intragastric administration of glucose fails to elevate insulin over the 5 min time period [41].

### 2.5. Measuring Fluid and Chow Consumption

Mice were offered two fluids per day in bottles. The control mice received two bottles with water, while the experimental mice received one bottle with water and another with an 11% sugar solution. Daily intake from each bottle was quantified (to the nearest 0.1 g) by measuring its change in weight over the previous 23.5 h with an analytical balance interfaced to a computer. Fluid spillage was estimated by measuring the change in weight of bottles containing the same fluids in an empty cage. The estimated spill was subtracted from the amount consumed over each 23.5 h period. We excluded water consumption from the data analysis because mice on the experimental diets drank the sugar solutions almost exclusively.

Chow intake was measured during the last two days of exposure period 3. On each day, each mouse was placed in a cage with fresh bedding and ~15 g of chow. We measured the change in weight of the chow across each day. Afterwards, we sifted the cage bedding by passing it through two sieves (grid sizes = 2.3 × 2.3 mm and 1.5 × 1.5 mm). We subtracted the weight of the recovered chow from our daily intake measurements.

### 2.6. Avidity for Sweeteners

**Figure 1:**
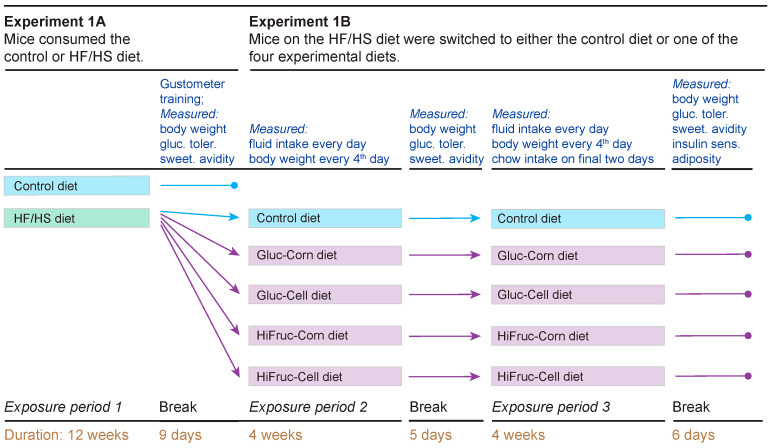
Experimental timeline. For Experiment 1A, mice were exposed to the control (n = 8) or HF/HS (n = 40) diet for 12 weeks. Following this exposure period (henceforth, exposure period 1), the mice received a 9-day break. After the break, the mice on the control diet were removed from the experiment. For Experiment 1B, the 40 mice on the HF/HS diet were randomly assigned to a control diet or one of four experimental diets. The control diet consisted of standard chow + water, and the experimental diets consisted of standard chow + water + an 11% solution of the Gluc-Corn, Gluc-Cell, HiFruc-Corn or HiFruc-Cell syrup. The mice received these diets for two 4-week exposure periods (i.e., exposure periods 2 and 3). There was a 5-day break between exposure periods 2 and 3, and a 6-day break after exposure period 3. During each break, the mice were fed standard chow and water, unless indicated otherwise. We took multiple measurements from each mouse at various times across the experiment. The measurements included body weight, glucose tolerance, sweetener avidity, daily fluid intake, daily chow intake, insulin sensitivity, and body composition. We indicate the approximate timing of each measurement above. The exact timing of each measurement is provided in Section 2.

We measured licking responses to six concentrations of sucrose (0.03, 0.1, 0.2, 0.3, 0.6, and 1M) and five concentrations of sucralose (0.3, 1, 3, 10, and 30 mM) in the gustometer, using brief-access lick tests [45]. We included water in each stimulus array, but did not report licks for the water because the numbers were extremely low. Each lick test lasted 30 min. A trial began when the mouse initiated licking from the sipper tube, and ended 5 s later when the shutter closed. After a 7.5 s pause, the mouse received a different solution. In this manner, the mouse could initiate up to 144 trials across the test. We treated the different concentrations of each sweetener (plus water) as a block, and randomized (without replacement) the order of presentation within the block so that each concentration was presented once before the next block began. We tested each sweetener on different days. The lick tests with sucrose and sucralose were conducted at three time-points: baseline (i.e., after Experiment 1A), after exposure period 2, and after exposure period 3 (Figure 1).

During the break between exposure periods 1 and 2 (Figure 1), the mice were subjected to three training sessions in the gustometer with water. These sessions served to familiarize each mouse with the gustometer and train them to drink fluids in it. A training session began when the mouse took its first lick, and it lasted 30 min. On day 1, each mouse was allowed to drink freely from the same sipper tube throughout the session. On days 2 and 3, the mouse could only drink water during sequential 5 sec trials. To motivate drinking, the mice were water-deprived for 22.5 h prior to each training session. Once a test session ended, the mouse was returned to its home cage and given 1 h access to food and water; then, it was water-deprived for another 22.5 h.

We motivated licking for the sucrose and sucralose solutions by food- and water-restricting the mice. This involved placing each mouse in a cage with fresh bedding 23.5 h prior to each test session, and limiting it to 1 g of chow (F0173; Bio-Serv, Flemington, NJ, USA) and 2 mL of water. This deprivation schedule has been found to cause B6 mice to exhibit robust concentration-dependent increases in lick rate for preferred taste stimuli during brief-access lick tests [45].

### 2.7. Insulin Tolerance Test (ITT)

At the start of the ITT, we measured each mouse’s weight and blood glucose concentration. Then, we injected insulin (Humulin R; Lilly, Indianapolis, IN, USA; lilly.com) intraperitoneally at a dose of 1 U/kg body weight. We subsequently transferred the mouse to a fresh cage lacking food and water, and measured blood glucose 5, 15, 30, and 60 min after the injection. Each ITT was conducted between 12:00 and 3:00 p.m. The mice were food- and water-replete prior to the test.

### 2.8. Body Weight and Percentage Body Fat Measurements

We weighed mice (to the nearest 0.1 g) on an analytical balance. After exposure period 3, we determined the body composition of each mouse using a micro-CT imaging system (PerkinElmer Quantum FX, Waltham, MA, USA; content.perkinelmer.com).

### 2.9. Experimental Timeline

#### 2.9.1. Experiment 1A

During exposure period 1, we randomly assigned age-matched mice to the HF/HS diet (HF/HS chow + water) (n = 40) or the control diet (standard chow + water) (n = 8) for 12 weeks. This exposure period was followed by a 9-day break, during which the mice received the control diet, unless indicated otherwise (Figure 1). On day 1 of the break, body weight was measured; on days 2–4, the mice were trained to lick in the gustometer; on day 5, the mice were subjected to a GTT; and on days 6 and 8, the mice were subjected to a brief-access lick test with sucralose and sucrose, respectively. Day 9 was a recovery day. Afterwards, the 40 mice on the HF/HS diet were integrated into Experiment 1B, while the eight mice on the control diet were removed from the experiment.

#### 2.9.2. Experiment 1B

We randomly assigned the 40 mice on the HF/HS diet to one of five diets (n = 8 mice/diet; Figure 1). They included a control diet (standard chow and water) and four experimental diets (standard chow, water and an 11% solution of the Gluc-Corn, Gluc-Cell, HiFruc-Corn, or HiFruc-Cell syrup). We balanced the sex ratio of mice on each diet. We measured fluid intake daily and body weight every 4 days across exposure periods 2 and 3. On the final two days of exposure period 3, we measured the daily intake of the standard chow.

There was a 6-day break between exposure periods 2 and 3. On day 1 of the break, mice were run through a GTT; and on days 2 and 4, mice were subjected to a brief-access lick test with sucralose and sucrose, respectively.

There was another 6-day break after exposure period 3. On day 1 of the break, the mice were run through a GTT; on days 2 and 4, the mice were subjected to a brief-access lick test with sucralose and sucrose, respectively; and on day 6, the mice were run through the ITT and a body composition analysis.

### 2.10. Data Analysis

All statistical analyses were conducted with Prism 10 (graphpad.com). We used the Shapiro–Wilk test to evaluate the data for normality. When data violated the normality assumption, we ran nonparametric tests. For ANOVAs with repeated factors, we used the Greenhouse–Geisser correction. In all analyses, we set alpha at 0.05. We describe the statistical procedures in Section 3 and the figure and table legends.

We measured blood glucose responses to the GTTs in three ways. First, we used blood glucose concentration at the 0 min time-point of the GTT as an estimate of fasting glucose. Second, we examined glucose dynamics across the GTT. Third, to obtain a more integrated measure of the glucose response, we calculated the area under the glucose response curves.

We examined plasma insulin dynamics across the GTT in four ways. To test for dietinduced changes in fasting insulin levels, we examined insulin levels at the 0 min time-point of the GTT. Second, we examined changes in absolute insulin concentrations across the GTT. Third, we divided the GTT into cephalic (i.e., 0–5 min) and postprandial (i.e., 15–60 min) phases, and analyzed results in each phase separately. Finally, to control diet-related differences in fasting insulin levels, we used a relative insulin response [46]. To this end, we determined the % increase in insulin concentration across the GTT, relative to that at the 0 min time-point.

We tested for diet-induced changes in avidity for sweetener solutions by examining two measures. The number of trials initiated during the 30 min test session reflected the extent to which mice repeatedly initiated licking for the sweetener solutions. As such, they provided an indirect measure of motivation to obtain these solutions [47,48,49]. Lick rates provided a measure of a solution’s hedonic value—the higher the lick rate, the higher the palatability [50]. To normalize lick rates across mice, we used a standardized lick ratio (SLR) [45]. To this end, we first determined each mouse’s local lick rate (in licks/s) during the first training session. Next, we determined the mean inter-lick interval (ILI), using the population of ILIs < 200 ms. We used the reciprocal of the mean ILI to estimate the local lick rate. Next, we multiplied the local lick rate by a scaling factor of 5, producing an estimate of the maximal number of licks that the mouse could complete if it licked continuously across the 5 s trial. Finally, the SLR was calculated by dividing the mean number of licks per trial (for a specific solution) by the maximal number of licks the same mouse could complete per trial. An SLR approaching 0.0 indicated that the sweetener solution elicited only sporadic licking, whereas an SLR approaching 1.0 indicated that it elicited nearly continuous licking. To facilitate comparisons across exposure periods, we calculated the area under the SLR concentration-response curves, separately for each mouse and sweetener.

## 3. Results

### 3.1. Experiment 1A: Impact of Exposure to the HF/HS Diet

**Figure 2:**
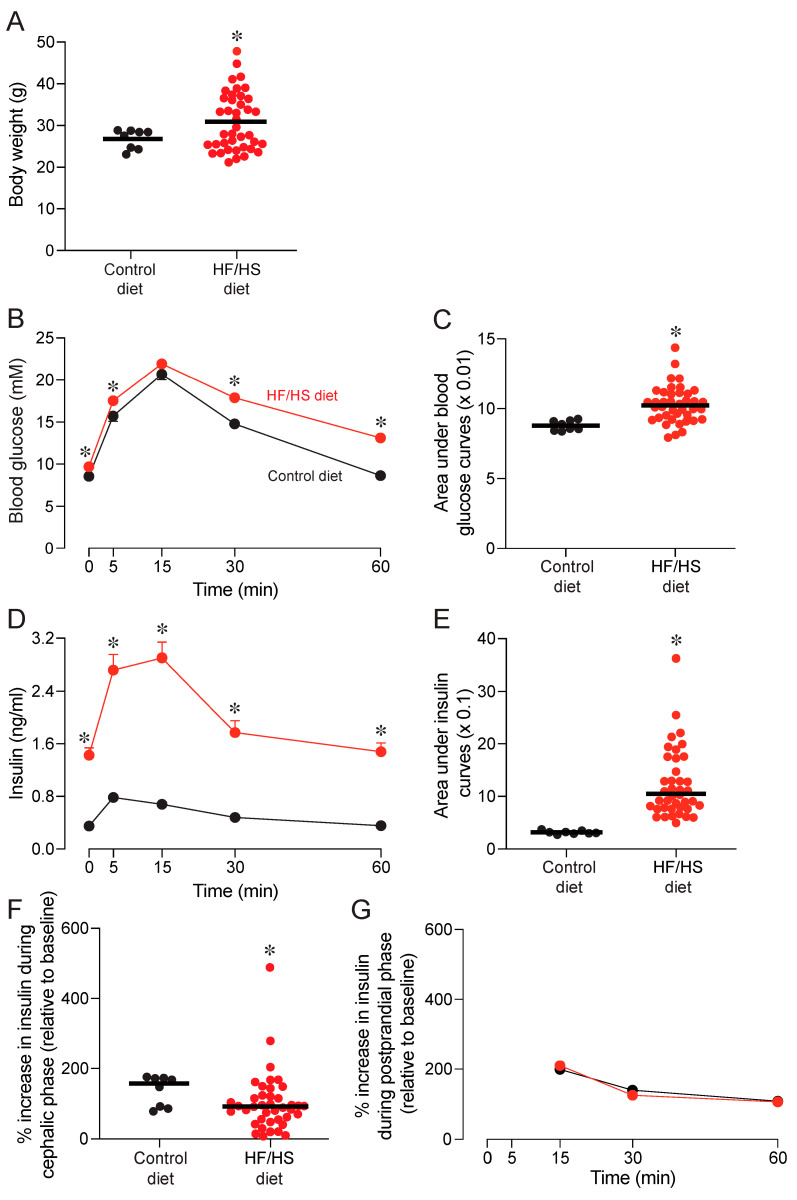
Experiment 1A. Body weights and metabolic status of mice on the control versus HF/HS diet, at the end of exposure period 1. Within each panel, we juxtapose responses to the two diets: (**A**) body weights, (**B**) blood glucose dynamics (mean ± SE) across the GTT, (**C**) area under the glucose response curves, (**D**) insulin dynamics across the GTT, (**E**) area under the insulin response curves, and % increase in insulin levels, relative to baseline, across the (**F**) cephalic (i.e., 0–5 min) and (**G**) postprandial (i.e., 15–60 min) phases of the GTT. In (**A**,**C**,**E**,**F**), we compare metabolic responses to the HF/HS and control diets with Mann–Whitney U tests (* *p* < 0.05). In (**B**,**D**), we indicate the time-points at which means for mice on the HF/HS diet were greater than those for mice on the control diet, according to Šídák’s multiple comparison test (* *p* < 0.05). In (**A**,**C**,**E**,**F**), we indicate the responses of individual mice with a circle, and medians with a horizontal line. n = 40 mice on HF/HS diet, and n = 8 mice on control diet.

To confirm that 12 weeks of exposure to the HF/HS diet induced symptoms of metabolic syndrome, we made several comparisons between the mice on the HF/HS versus control diet. The body weights of mice on the HF/HS diet were >8 g higher than those of the mice on the control diet (unpaired t-value = 3.7, *df* = 46, *p* = 0.0006) (Figure 2A).

We analyzed two features of the glycemic response to the GTT. Fasting blood glucose levels (i.e., those collected at the 0 min time-point of the GTT) on the HF/HS diet were larger than those on the control diet (unpaired t-value = 2.76, *df* = 46, *p* = 0.0082) (Figure 2B). A mixed-model ANOVA of glucose responses across the GTT revealed a main effect of diet (F_1, 46_ = 12.8, *p* = 0.0008) and time (F_2.6, 121.4_ = 101.4, *p* < 0.001), but no interaction of diet × time (F_4, 184_ = 1.3, *p* > 0.27). These results were corroborated by an analysis of the area under the glucose response curves, which revealed greater area in mice on the HF/HS than the control diet (Figure 2C). It follows that consumption of the HF/HS diet impaired glucose tolerance.

We also examined several features of insulin response. The fasting insulin levels of mice on the HF/HS diet were higher than those of mice on the control diet (unpaired t-value = 4.3, *df* = 46, *p* < 0.0001) (Figure 2D). A mixed-model /ANOVA of the insulin responses across the GTT revealed a main effect of diet (F_1, 46_ = 17.1, *p* = 0.0001) and time (F_2.5, 114.4_ = 16.1, *p* < 0.0001), and an interaction of diet × time (F_4, 184_ = 5.5, *p* = 0.0003). This result, together with the analysis of the area under the insulin response curves (Figure 2E), reveals that consumption of the HF/HS diet increased the size of the insulin response.

The foregoing analysis did not provide clear insight into whether consumption of the HF/HS diet caused the mice to generate enhanced insulin responses during the GTT. This is because the fasting insulin concentration at the onset of the GTT was roughly five times larger in mice on the HF/HS diet than the control diet. To control for this initial difference, we calculated the % increase in insulin concentration relative to the 0 min time-point of the GTT. During the initial 5 min (i.e., cephalic phase) of the GTT, the relative insulin response was slightly, but significantly, lower in mice on the HF/HS diet (Mann–Whitney U-value = 80.5, *p* < 0.03) (Figure 2F), revealing that the HF/HS diet diminished CPIR magnitude. During the 15–60 min (i.e., postprandial phase) of the GTT, there was a main effect of time (F_1.5, 71.1_ = 40.4, *p* < 0.0001), but not of diet (F_1, 46_ < 0.1, *p* = 0.89) or interaction of time × diet (F_2, 92_ = 1.1, *p* < 0.32) on the % increase in insulin concentration (Figure 2F). This shows that the HF/HS diet had no effect on the postprandial insulin response to the GTT.

**Figure 3:**
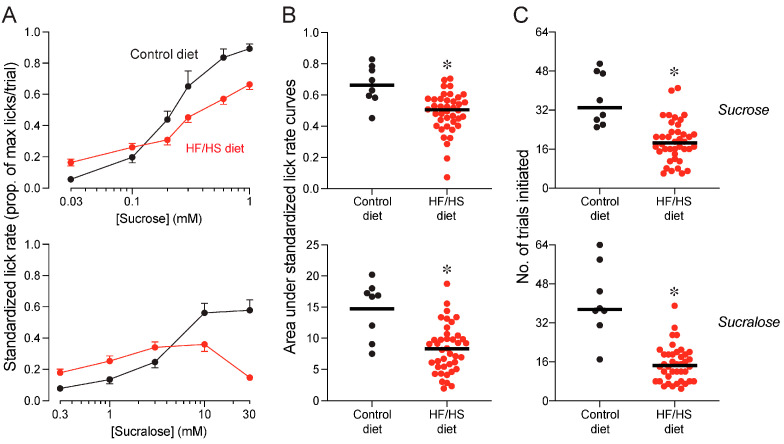
Experiment 1A. Avidity for sucrose (top row of panels) and sucralose (bottom row of panels) in mice on the control or HF/HS diet, at the end of exposure period 1. We show (**A**) standardized lick rates (SLRs) across 5–6 concentrations of sucrose and sucralose (mean ± S.E.), (**B**) area under the concentration-response functions for each sweetener, and (**C**) the number of trials initiated for each sweetener. In (**B**,**C**), we compare the responses of mice on the HF/HS and control diets, using Mann–Whitney U tests (* *p* < 0.05). We indicate the responses of individual mice with a circle, and median responses with a horizontal bar. n = 40 mice on HF/HS diet, and n = 8 mice on control diet.

Next, we asked how the HF/HS diet impacted avidity for the sucrose and sucralose solutions. To this end, we compared lick rates (as indicated by SLRs) and numbers of trials initiated for the two sweeteners between mice on the HF/HS versus control diet. Lick rates for sucrose increased with concentration in mice on both diets, but it increased to a lesser extent in mice on the HF/HS diet (Figure 3A, upper panel). This inference is supported by less area under the concentration-response curves for sucrose in mice on the HF/HS diet relative to mice on the control diet (unpaired t-value = 3.6, *df* = 46, *p* = 0.0008; Figure 3B, upper panel). In addition, mice on the HF/HS diet initiated fewer trials for the sucrose solutions than did mice on the control diet (unpaired t-value = 5.0, *df* = 46, *p* < 0.0001; Figure 3C, upper panel). Taken together, these results show that consumption of the HF/HS diet reduced avidity for sucrose.

Exposure to the HF/HS diet caused even more profound reductions in avidity for the sucralose solutions. Mice on the control diet exhibited concentration-dependent increases in lick rate for sucralose, whereas mice on the HF/HS diet did not (Figure 3A, lower panel). This resulted in substantially less area under the concentration-response curves for mice on the HF/HS diet (unpaired t-value = 4.2, *df* = 46, *p* < 0.0001; Figure 3B, lower panel). Further, mice on HF/HS diet initiated less than half as many trials for the sucralose solutions as did mice on the control diet (unpaired t-value = 7.4, *df* = 46, *p* < 0.0001; Figure 3C, lower panel).

#### Sex Effects

For the analyses described above, we collapsed across sex. Here, we tested for an effect of sex on the metabolic and behavioral responses of mice on the HF/HS or control diet. Irrespective of diet, males weighed more than females at the end of exposure period 1 (Figure S1A). The sex difference in body weight, however, was more pronounced in mice on the HF/HS diet. Sex did not impact glycemic responses of mice to the GTT (Figure S1B; Table S1), but it did impact insulin responses (Figure S1C; Table S1). The males on the HF/HS diet exhibited larger insulin responses than females at all time-points across the GTT; there was no corresponding sex difference in mice on the control diet.

There was no main effect of sex on standardized lick rates for the different concentrations of sucralose (Figure S1D; Table S2) or sucrose (Figure S1F; Table S2). Likewise, there was no effect of sex on the number of trials initiated during the brief-access lick tests with sucrose or sucralose (Figure S1E,G).

Taken together, the results of Experiment 1A show that 3 months of exposure to the HF/HS diet caused weight gain, glucose intolerance, hyperinsulinemia (particularly in males), lower CPIR magnitudes, and reduced avidity for sweeteners. In Experiment 1B, we asked whether switching these metabolically deranged mice to a control or one of the four experimental diets would improve these metabolic and behavioral measures.

### 3.2. Experiment 1B: Impact of Switching Mice to the Control or Experimental Diets

#### 3.2.1. Weight Changes and Daily Intake

**Figure 4:**
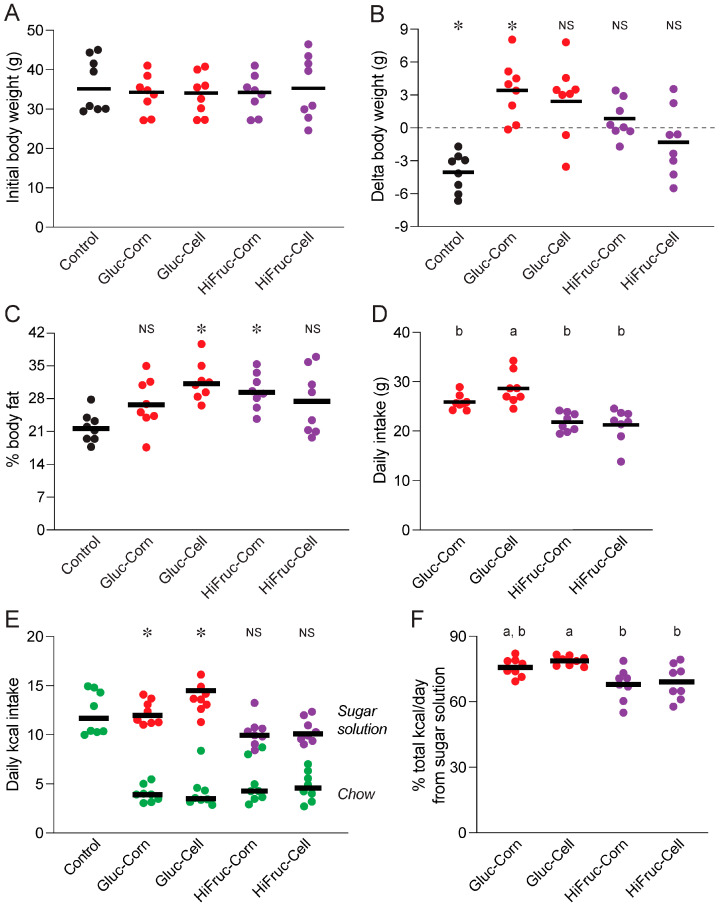
Experiment 1B. (**A**) Body weights on mice on the different diets at baseline (i.e., the end exposure period 1). (**B**) Changes in body weight across exposure periods 2 + 3, relative to baseline. For each diet, we compared the delta weight values to zero, using a one-sample *t*-test (*NS*, *p* ≥ 0.05, * *p* < 0.05). (**C**) The % body fat of mice on the different diets at the end of exposure period 3. (**D**) Daily intakes (in g) of the Gluc-Corn, Gluc-Cell, HiFruc- Corn, or HiFruc-Cell solutions across exposure periods 2 + 3. (**E**) Daily kcal intake/day from the chow versus each of sugar solutions during the final two days of exposure period 3. We compare total calories ingested/day from the control diet (i.e., chow only) with those ingested/day from each of the experimental diets (i.e., chow + sugar solution), using Dunnett’s multiple comparison test (*NS*, *p* ≥ 0.05, * *p* < 0.05). (**F**) Percent of total kcal obtained/day from sugar syrup in each of the experimental diet, during the final two days of exposure period 3. In all panels, we represent responses of each mouse with a circle, and mean responses with a horizontal bar. In (**B**,**C**,**E**), we compare mice on the control versus experimental diets with Dunnett’s multiple comparison test (*NS*, *p* ≥ 0.05, * *p* < 0.05). In (**D**,**F**), we compare responses to each diet treatment with Tukey’s multiple comparison test; different letters (a, b) indicate diet treatments that differ from one another (*p* < 0.05). n = 8 mice per diet treatment.

At the beginning of exposure period 2, there were no differences in body weight across all five diet treatments (F_4, 35_ = 0.33, *p* = 0.86; Figure 4A). To test for diet-induced changes in body weight, we calculated the difference in body weight between the start of exposure period 2 and end of exposure period 3, separately for each mouse. Then, we compared these delta values to zero, separately for each diet treatment, using one-sample *t*-tests (Figure 4B). Mice on the control diet lost an average of 4.0 g, whereas mice on the Gluc-Corn diet gained an average of 3.4 g. Mice on the Gluc-Cell, HiFruc-Corn, or HiFruc-Cell diets did not experience any systematic change in weight.

There were diet-related differences in adiposity (% body fat) at the end of exposure period 3 (F_4, 35_ = 4.6, *p* = 0.005) (Figure 4C). As compared with mice on the control diet, those on the Gluc-Cell and HiFruc-Corn diets had higher adiposity and those on the Gluc-Corn and HiFruc-Cell diets had similar adiposity. In a separate analysis, we examined the effect of sex on body composition at the end of exposure period 3 (Table S2). For % body fat (adiposity), there was no effect of sex or interaction between sex × diet. By contrast, for total body fat and lean mass, there was a significant main effect of sex, but no interaction of diet × sex. These analyses reveal that male and female mice had similar adiposity, but because males were larger, they had more overall body fat.

We analyzed daily intake of the sugar solutions and chow in several ways. First, we determined the mean amount of sugar solution consumed per day across exposure periods 2 and 3. We found that mice consumed more of the Gluc-Cell solution than of the Gluc-Corn, HiFruc-Corn, or HiFruc-Cell solutions (F_3, 28_ = 13.2, *p* < 0.0001) (Figure 4D). We also calculated daily kcal intake from chow and each sugar solution during the final two days of exposure period 3. On these days, mice on the experimental diets consumed large amounts of each sugar solution and relatively small amounts of chow (Figure 4E). To determine whether this feeding response effectively limited daily kcal intake to levels comparable to those of mice on the control diet, we compared the total kcal intake per day by mice on the control diet (chow only) and experimental diets (chow + 11% sugar solution). A one-way ANOVA (F_4, 35_ = 6.0, *p* < 0.001) revealed significant differences across diets. Mice on the Gluc-Corn and Gluc-Cell diets ingested more total kcals per day than mice on the control diet, whereas mice on the HiFruc-Corn and HiFruc-Cell diets ingested similar kcals per day as mice on the control diet. This shows that mice on the two HiFruc diets limited total kcal per day to that of mice on the control diet, while mice on the two Gluc diets consumed more total kcal per day than control mice.

Mice on the experimental diets obtained a large percentage (68–79%) of their total kcal per day from the sugar solutions, as opposed to chow, during the final two days of exposure period 3 (Figure 4F). A one-way ANOVA revealed systematic differences across the experimental diets (F_3, 28_ = 6.1, *p* < 0.003). The mean % of daily kcals derived from the Gluc-Cell (79%) syrup exceeded that from the HiFruc-Corn (68%) and HiFruc-Cell (69%) syrups. In contrast, the mean % of daily kcals derived from the Gluc-Corn (76%) syrup, however, did not differ from that from the other syrups.

To determine the stability of daily sugar intake across exposure periods 2 and 3, we compared the daily intake of each sugar solution both within and between exposure periods, using two-way repeated measures ANOVAs (see Figure S2, Table S3). For all four sugar solutions, there was a significant main effect of exposure period; this reflected greater daily intake of each sugar solution during exposure period 3 than exposure period 2. Further, there was a significant interaction for the Gluc-Corn and Gluc-Cell solutions, but not for the other two sugar solutions. These interactions reflect the fact that the mice initially consumed relatively low quantities of the Gluc-Corn and Gluc-Cell solutions during days 1–3 of exposure period 2, but rapidly increased daily intake. This same pattern was not observed during exposure period 3.

**Figure 5:**
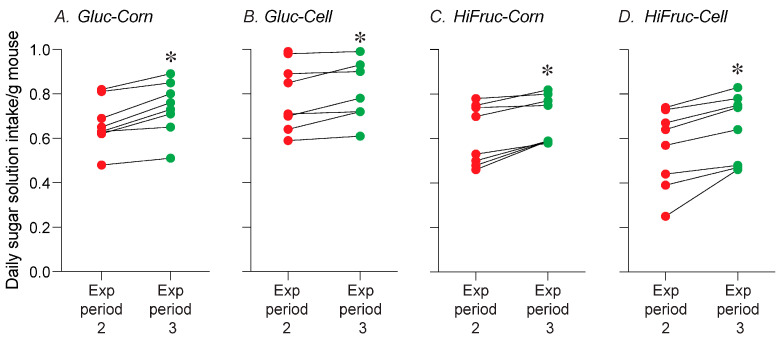
Experiment 1B. Daily intake of the (**A**) Gluc-Corn, (**B**) Gluc-Cell, (**C**) HiFruc-Corn, or (**D**) HiFruc-Cell solutions increased across exposure period 2 and 3. We normalized each mouse’s daily intake values to body weight, and then collapsed the normalized values across time, separately for each exposure period. Within each panel, we compare values (i.e., mean daily intake/g mouse) across exposure periods 2 and 3, using paired tests (* *p <* 0.05). We indicate values from each mouse with two circles connected by a line. See Figure S2 and Table S3 for the presentation and analysis of raw daily intake values (i.e., measurements that have not been collapsed over time or normalized to body weight). n = 8 mice per diet.

To confirm that the higher intake of the sugar solutions during exposure period 3 did not reflect higher body weights of the mice, we normalized daily intake of each sugar solution to each mouse’s body weight, and then re-analyzed the data. To this end, we first calculated the mean normalized daily intake/mouse for each sugar solution, separately for exposure period 2 and 3. Then, we compared these normalized daily intake values across exposure periods with paired *t*-tests. We found that the normalized daily intake values during exposure period 3 were higher than those during exposure period 2 for all of the sugar solutions (Figure 5A–D). This finding confirms that daily intake of the sugar solution increase across exposure periods 2 and 3.

#### 3.2.2. Glucose Tolerance and Insulinemia

**Figure 6:**
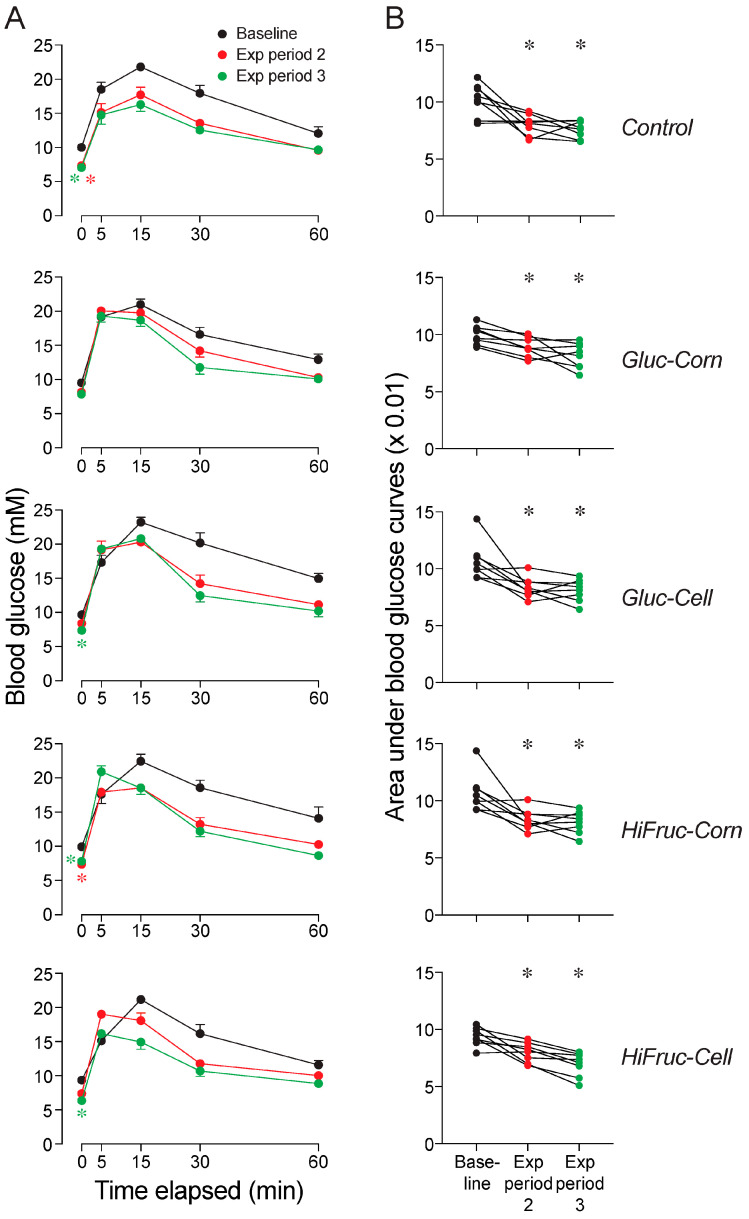
Experiment 1B. Switching the mice to the control or experimental diets caused glucose tolerance to recover. Each panel contains glycemic responses to the oral GTT at three time-points: baseline and after exposure periods 2 and 3. We show (**A**) blood glucose dynamics (mean ± SE) across the GTT and (**B**) area under the glucose response curves. Within each row of panels, we present results from mice on the control or one of the experimental diets. In (**A**), we asked whether any of the diets decreased fasting glucose levels. To this end, we compare fasting blood glucose values at baseline with those at the end of exposure periods 2 and 3, using Dunnett’s multiple comparison test (* *p <* 0.05). We use color to distinguish asterisks that reflect lower fasting glucose levels after exposure period 2 (red) or exposure period 3 (green). In (**B**), we compare the area under the glucose response curves at baseline with those after exposure periods 2 and 3, using Dunnett’s multiple comparison test (* *p <* 0.05). We indicate values from each mouse with three circles connected by a line. See Table S5 for further analysis of these data. n = 8 mice per diet.

We asked how switching mice from the HF/HS diet to the control or experimental diets impacted glucose tolerance. To this end, we examined the GTT results collected at three time-points (baseline, and the end of exposure periods 2 and 3), separately for each diet (Figure 6). We found that fasting blood glucose levels decreased across exposure periods 2 and 3 on all diets, except the Gluc-Corn diet (Figure 6A). Second, the magnitude of the glycemic responses of the mice on the control and experimental diets decreased across exposure periods 2 and 3 (Figure 6A, Table S5). This finding was reinforced by parallel reductions in the area under the glycemic response curves across exposure periods 2 and 3 (Figure 6B).

**Figure 7:**
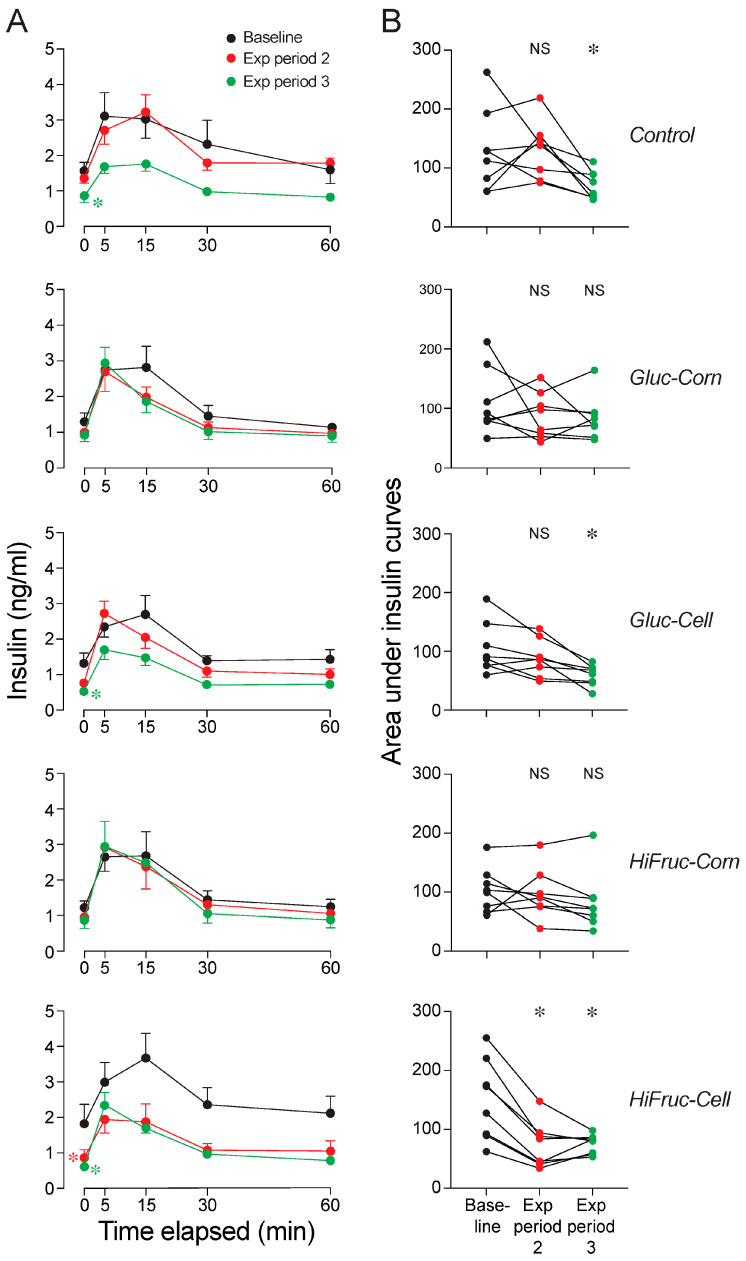
Experiment 1B. Switching mice to the control, Gluc-Cell, or HiFruc-Cell diets reduced insulinemia. We juxtapose insulin responses to the oral GTT from three time-points: baseline and after exposure periods 2 and 3. In (**A**), we show insulin dynamics (mean ± SE) across the GTT, separately for each of the diets. In (**B**), we show the area under the insulin response curves. To determine whether the any of the diets reduced fasting insulin levels, we compared insulin values at the 0 min time-point at baseline with those after exposure periods 2 and 3, using Dunnett’s multiple comparison test (* *p <* 0.05). We use color to distinguish asterisks that reflect lower fasting insulin levels after exposure period 2 (red) or 3 (green). (**B**) We also compare the area measurements at baseline with those after exposure periods 2 and 3, using Dunnett’s multiple comparison test (*NS*, *p* ≥ 0.05; * *p <* 0.05). We represent values from each mouse with three circles connected by a line. See Table S5 for further analysis of these data. n = 8 mice per diet.

Next, we examined the impact of the experimental diets on insulin responses to the GTT. We found that fasting insulin levels (Figure 7A), magnitude of the insulinemic responses (Figure 7A; Table S5), and area under the insulin response curves (Figure 7B) all decreased over exposure periods 2 and 3 in mice on the control, Gluc-Cell, and HiFruc-Cell diets. In contrast, none of these measures changed in mice on the Gluc-Corn or HiFruc-Corn diets. This shows that consumption of the control and experimental diets with cellulosic sugars reduced insulinemia, but consumption of experimental diets with corn sugars had no impact on insulinemia. by extension, these results also indicate that consumption of the corn syrups actually impeded recovery of insulinemia.

**Figure 8:**
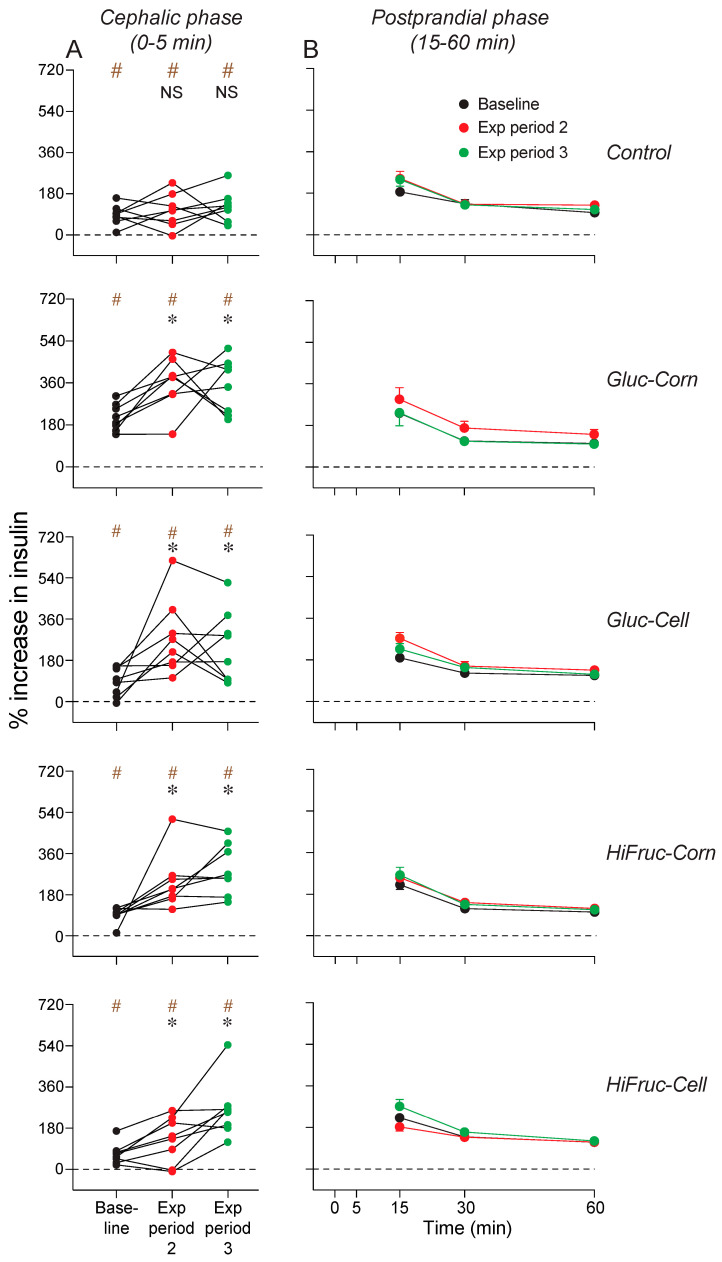
Experiment 1B. Percent increase in insulin concentration during the (**A**) cephalic and (**B**) postprandial phases of the GTT. Within each row of panels, we present results from mice on the control or one of the experimental diets. In (**A**), we ask whether the mice exhibited a CPIR by determining whether the mean % increase in insulin levels, relative to that at the 0 min time-point, is greater than zero (# *p* < 0.05, one-sample *t*-test; symbols are brown). We perform these *t*-tests separately one results collected at baseline and after exposure periods 2 and 3. In addition, we compare CPIR magnitudes at baseline with those after exposure periods 2 and 3, using Dunnett’s multiple comparison test (*NS*, *p* ≥ 0.05; * *p <* 0.05; symbols are black). We indicate the responses of each mouse with circles connected by a line. In (**B**), we show % increase in insulin concentration at the 15, 30 and 60 min time-points of the GTT, relative to the 0 min time-point (mean ± SE). In each panel, we show results collected at baseline and after exposure periods 2 and 3. See Table S6 for the analysis of these results. n = 8 mice per diet.

Finally, we examined changes in insulin concentration across the cephalic (0–5 min) (Figure 8A) and postprandial (15–60 min) (Figure 8B) phases of the GTT. To this end, we determined the % increase in insulin concentration at the 5, 15, 30 and 60 min time-points of the GTT, relative to that at the 0 min time-point. The mice on the control and experimental diets all exhibited a CPIR (i.e., a significant % increase in insulin concentration during the initial 5 min of the GTT) at baseline and at the end of exposure periods 2 and 3 (Figure 8A). When we tested for changes in CPIR magnitude over time, we found that CPIR magnitude was higher after exposure periods 2 and 3 (relative to baseline) in mice on the experimental diets, but not in mice on the control diet (Figure 8A). These results establish that consumption of the experimental diets increased CPIR magnitude, independent of any differences in fasting insulin levels.

We also asked whether the insulin response during the postprandial phase of the GTT changed across Experiment 1B, separately for each diet (Figure 8B; Table S6). However, the results contradicted this possibility. We found that postprandial insulin responses overlapped extensively at baseline and after exposure periods 2 and 3. Taken together, our results show that chronic consumption of the experimental diets induced larger insulin responses during the cephalic phase, but not the postprandial phase, of the GTT.

**Figure 9:**
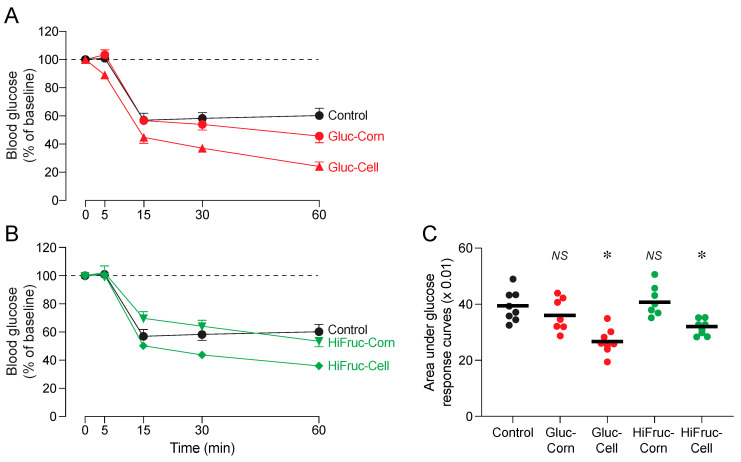
Experiment 1B. Response of mice on the control versus experimental diets to the insulin tolerance test (ITT). The ITT was conducted at the end of exposure period 3. We obtained an initial glycemic measurement at the 0 min time-point, and then administered an intraperitoneal injection of 1 MU/g of insulin. We subsequently measured blood glucose levels at the 5, 15, 30, and 60 min time-points (mean ± SE). All blood glucose values were normalized to those collected at the 0 min time-point (i.e., 100%). In (**A**), we juxtapose results from mice on the control, Gluc-Corn, and Gluc-Cell diets; in (**B**), we juxtapose results from mice on the control, HiFruc-Corn, and HiFruc-Cell diets; and in (**C**), we compare the area under the ITT curves in mice on the control diet with those of mice on each of the experimental diets, using Dunnett’s multiple comparison test (*NS, p* > 0.05, * *p* ≤ 0.05). The closed circles indicate the area under the ITT curve for each mouse, and the horizontal lines indicate means. n = 8 mice per diet.

We conducted an ITT at the end of exposure period 3 to determine whether the experimental diets altered insulin sensitivity. We found that mice on diets with cellulosic syrups (Gluc-Cell or HiFruc-Cell) had greater insulin sensitivity than mice on the control diet, whereas mice on diets with corn syrups (Gluc-Corn or HiFruc-Corn) had the same insulin sensitivity as mice on the control diet (Figure 9A–C). It follows that the consumption of the cellulosic syrups enhanced insulin sensitivity. This finding may help explain how mice on the Gluc-Cell and HiFruc-Cell diets were able to reduce insulin production, but still exhibit robust glucose tolerance, during the GTTs (see Figure 6 and Figure 7).

#### 3.2.3. Avidity for Sucrose and Sucralose

**Figure 10:**
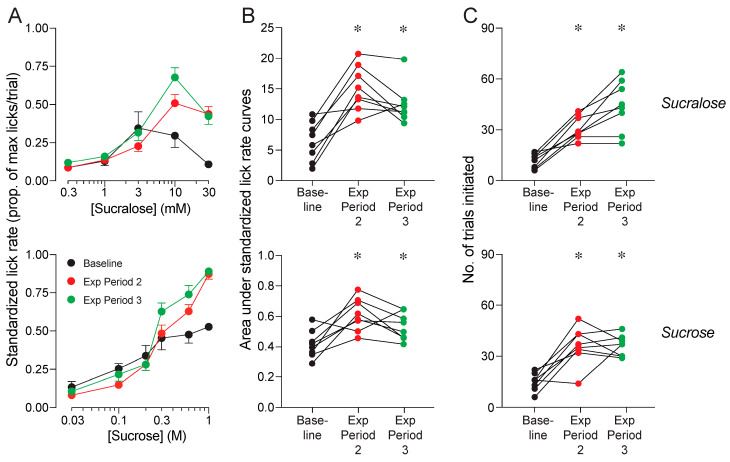
Experiment 1B. Avidity for sucralose (top row of panels) and sucrose (bottom row of panels) recovered when mice were switched to the control diet. We present three measures of avidity: (**A**) standardized lick rates for different concentrations of each sweetener (mean ± SE), (**B**) area under the concentration-response curves, and (**C**) the number of trials initiated per test session. Within each panel, we present responses collected at three time-points: baseline (i.e., after exposure period 1) and after exposure periods 2 and 3. In the panels within columns (B,C), we represent the responses of the individual mice with circles connected by a line. We compare the values collected at baseline with those at later time-points, using Dunnett’s multiple comparison tests (* *p <* 0.05). n = 8 mice per diet.

**Figure 11:**
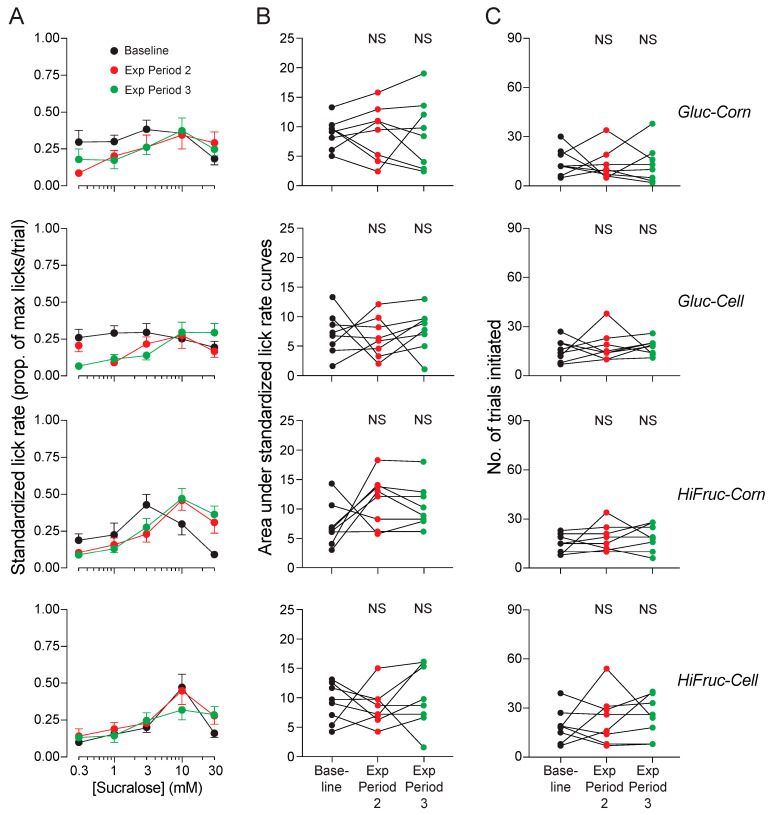
Experiment 1B. Avidity for sucralose did not recover when mice were switched to the Gluc-Corn, Gluc-Cell, HiFruc-Corn, or HiFruc-Cell diet. We show three measures of avidity: (**A**) standardized lick rates for different concentrations of sucralose (mean ± SE), (**B**) area under the concentration-response curves, and (**C**) the number of trials initiated per test session. We present results from each diet in separate rows of panels. Within each panel, we show responses collected at three time-points: baseline (i.e., after exposure period 1) and after exposure periods 2 and 3. In (**B**,**C**), we represent the responses of the individual mice with circles connected by a line. We compare the values collected at baseline with those at later time-points, using Dunnett’s multiple comparison tests (*NS*, *p* ≥ 0.05). n = 8 mice per diet.

**Figure 12:**
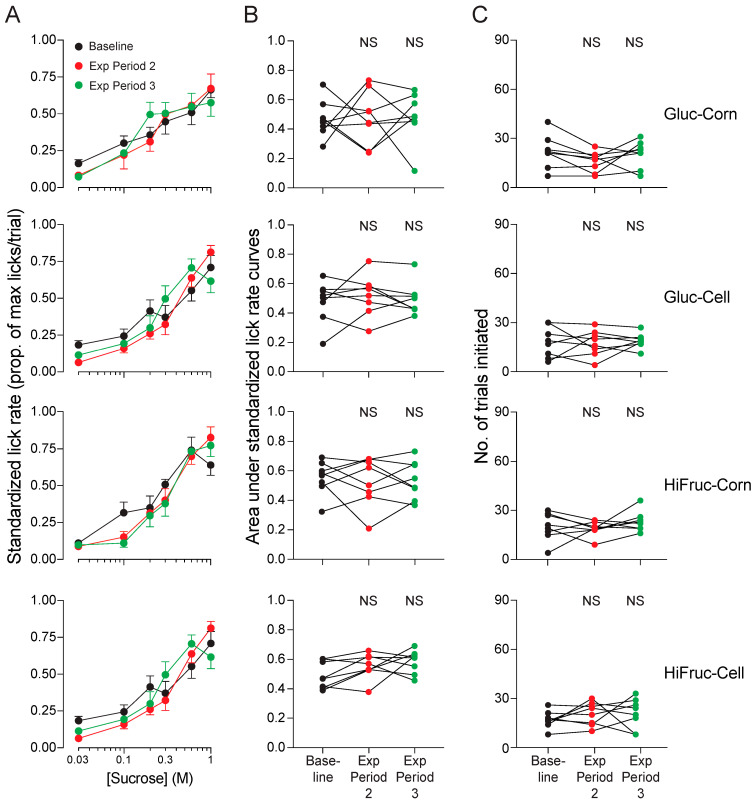
Experiment 1B. Avidity for sucrose did not recover in when mice were switched to the Gluc-Corn, Gluc-Cell, HiFruc-Corn, or HiFruc-Cell diet. We show three measures of avidity: (**A**) standardized lick rates for different concentrations of sucrose (mean ± SE), (**B**) area under the concentration-response curves, and (**C**) the number of trials initiated per test session. We present results from each diet in separate rows of panels. Within each panel, we show responses collected at three time-points: baseline (i.e., after exposure period 1) and after exposure periods 2 and 3. In (**B**,**C**), we represent the responses of the individual mice with circles connected by a line. We compare the values collected at baseline with those at later time-points, using Dunnett’s multiple comparison tests (*NS*, *p* ≥ 0.05). n = 8 mice per diet.

When the metabolically deranged mice were switched to the control diet, standardized lick rates and number of trials initiated for sucralose and sucrose increased across exposure periods 2 and 3 (Figure 10A–C). In fact, standardized lick rates at the end of exposure period 3 were comparable to those of the mice on the control diet in Experiment 1A, which lacked any prior exposure to the HF/HS diet (see Figure 3). In contrast, when the metabolically deranged mice were switched to the experimental diets, standardized lick rates and number of trials initiated for sucralose (Figure 11A–C) and sucrose (Figure 12A–C) remained low across exposure periods 2 and 3. These findings indicate that consumption of the experimental diets prevented the mice from regaining their avidity for sweeteners.

## 4. Discussion

### 4.1. Metabolic and Behavioral Effects of Exposure to the HF/HS Diet

During exposure period 1, the mice on the HF/HS diet developed higher body weights and fasting blood glucose levels than mice on the control diet. They also became glucose intolerant and hyperinsulinemic. This observation is consistent with prior findings [34,51,52,53] and reveals that chronic exposure to the HF/HS diet rendered the mice metabolically deranged.

Mice on the HF/HS diet also exhibited smaller CPIR magnitudes than mice on the control diet. This finding is consistent with a report that obese humans also tended to exhibit smaller relative CPIR magnitudes than normal-weight humans [46]. It is likely that the smaller CPIR magnitudes in mice on the HF/HS diet contributed to their impaired glucose tolerance [41,54].

Exposure to the HF/HS chow reduced avidity for the sucrose and sucralose solutions. This was manifested as (i) blunted concentration-dependent increases in lick rate for sucrose, (ii) the absence of concentration-dependent increases in lick rate for sucralose, and (iii) a large decrease in number of trials initiated for the sucrose and sucralose solutions. A prior study reported that consumption of a HF/HS diet reduced, but did not eliminate, concentration-dependent increases in lick rates for sucrose [10]. The extent to which lick rates for sucrose were reduced in the latter study, however, was more extreme than herein. This discrepancy may reflect methodological differences. For example, Johnson [10] tested the mice under water-deprivation and offered each sucrose concentration during separate 20 min test sessions, whereas we tested the mice under food and water restriction and offered each sucrose concentration during separate 5 s trials within the same 30 min test session. Another study reported that exposure to a high-fat diet eliminated concentration-dependent increases in lick rates for sucrose and two low-calorie sweeteners (acesulfame-K and saccharin) [55], but had no impact on the number of trials initiated for each sweetener. Again, the discrepant findings between Ahart et al. [55] and the present study may reflect methodological differences. Ahart et al. [55] exposed the mice to a high-fat diet and tested the mice under water-deprivation.

Why did exposure to the HF/HS diet eliminate concentration-dependent increases in lick rates for sucralose, but only blunt them for sucrose? This may stem from the fact that sucralose was inherently less attractive to mice, given that it elicited lower lick rates than sucrose in mice on the control diet (Figure 3). Lower lick rates are associated with lower sweet taste intensities [50]. Accordingly, exposure to the HF/HS diet may have diminished licking responses to the sucralose disproportionately because it was less attractive to the mice.

### 4.2. Impact of the Control Diet on Metabolism and Ingestive Behavior

Switching mice from the HF/HS to the control diet caused weight loss and marked improvements in fasting blood glucose and glucose tolerance. Indeed, the area under the glycemia curve after exposure periods 2 and 3 (Figure 6B) was similar to that observed in mice on the control diet after exposure period 1 (Figure 2B). Switching mice to the control diet also reduced insulinemia by the end of exposure period 3, but the level of insulinemia in these mice remained high. For instance, the mean fasting insulin levels of mice on the control diet at the end of exposure period 3 was ~0.9 ng/mL (Figure 7A), whereas that of mice on the control diet at the end of exposure period 1 was ~0.3 ng/mL (Figure 2D). It follows that insulin resistance persisted across exposure periods 2 and 3.

Switching mice to the control diet had no impact on CPIR magnitude. The % increase in plasma insulin across the cephalic phase of the GTT was roughly 100% at baseline and at the end of exposure periods 2 and 3. The stability of CPIR magnitudes over time contrasted with that of mice on the experimental diets (see below).

Finally, switching mice to the control diet caused them to regain their normal avidity for sucralose and sucrose by the end of exposure period 2. Indeed, standardized lick rates for sucrose and, to a lesser extent, sucralose, rose to a level comparable to those of mice on the control diet at the end of exposure period 1. This result is consistent with a prior report that metabolically deranged mice can recover their avidity for sweeteners within 10 days of switching to standard chow [10]. In contrast, there is another report (i) that 10 weeks of exposure to a HF/HS chow reduced the number of fungiform and circumvallate taste papillae in rats, and (ii) that switching the same rats to standard chow for 38 weeks did not cause the lost taste papillae to reappear [56]. However, because the latter study did not measure licking responses to sweeteners, it could not exclude the possibility that taste cells in the spared taste papillae were sufficient to mediate recovery of normal licking responses to sweeteners.

### 4.3. Impact of the Experimental Diets on Blood Glucose Control, Hyperinsulinemia, and Insulin Responses

Switching mice to the experimental diets caused recovery of glucose tolerance. Indeed, mice on all of the experimental diets exhibited marked improvement in glucose tolerance by the end of exposure periods 2 and 3. There are two important implications of this finding. The first stems from the observation that glucose tolerance recovered at the same rate in mice on the control and experimental diets. This indicates that the key factor driving recovery of glucose tolerance was simply removing the mice from the HF/HS diet. It also follows that the presence of the sugars in the experimental diets did not impede recovery of glucose tolerance. This latter finding is surprising given that the mice consumed such large quantities of the sugar solutions across exposure periods 2 and 3. Indeed, during the final two days of exposure period 2, sugars represented 68–79% of total calories consumed each day. More work is needed to reconcile this observation with the recommendation of the World Health Organization that “in both adults and children, the intake of free sugars should be reduced to less than 10% of total energy intake” [57] (p. 4).

Even though mice on the control and experimental diets recovered glycemic control, they did not do so in the same way. For example, mice on the Gluc-Cell diet experienced reductions in insulinemia after exposure period 2, while mice on the control and HiFruc-Cell diets did not do so until after exposure period 3. By contrast, mice on the Gluc-Corn and HiFruc-Corn diets did not experience any reductions in insulinemia after exposure periods 2 or 3.

Consumption of the experimental diets enhanced CPIR magnitudes. This finding is remarkable in light of the fact that the mice were already exhibiting extremely high fasting insulin levels, owing to prior maintenance on the HF/HS diet. Given that CPIR magnitudes reliably predict glucose tolerance in mice [44], it is likely that the higher CPIR magnitudes contributed to the improvements in glycemic control at the end of exposure periods 2 and 3.

How did the dietary sugars increase CPIR magnitude? The most parsimonious answer is that they increased the parasympathetic innervation of pancreatic islets, resulting in greater (or more effective) stimulation of the beta cells. We base this inference on three observations. First, CPIR is thought to be elicited by the parasympathetic stimulation of pancreatic beta cells, given that CPIR is abolished (i) in rats with denervated islets and (ii) in normal rodents treated with a drug (atropine) that blocks acetylcholine receptors [44,58,59]. Second, the afferent limb of the CPIR pathway appears to be activated by a glucose-specific taste pathway in mice [41,60,61], and the Gluc and Hi-Fruc diets all contained abundant glucose. Accordingly, consumption of the sugar solutions would have caused strong and repeated activation of the glucose-specific taste pathway. Third, a recent report revealed that mice with streptozotocin-induced diabetes or humans with type 2 diabetes exhibit enhanced innervation of the pancreatic islets [62]. Parenthetically, it is unlikely that the Gluc diets enhanced CPIR magnitude by increasing beta cell mass and/or insulin secretory activity. This is because the latter changes would be expected to have increased insulin secretion across the entire GTT, not just during the cephalic phase [63].

At the end of exposure period 3, the mice on diets with cellulosic sugars, but not corn sugars, exhibited better insulin sensitivity than did mice on the control diet. While more work is needed to explain this observation, it likely reflects, at least in part, the presence of polyphenols in the cellulosic syrups (e.g., gallic acid, protocatechuic acid, and 4-hydroxybenzoic acid, see Section 2.2). Indeed, prior studies revealed that the administration of polyphenols to metabolically deranged rodents markedly improves glucose tolerance and insulin sensitivity [26,28,31]. None of these studies, however, reported that the polyphenols caused insulin sensitivity to exceed that observed in control rodents. Thus, our results are notable because the polyphenol-containing cellulosic syrups induced greater insulin sensitivity than the control diet.

### 4.4. Impact of Experimental Diets on Sweetener Avidity and Daily Intake of Sugar Solutions

When mice were switched from the HF/HS diet to the experimental diets, they did not experience any recovery in avidity for the sucrose or sucralose solutions. Indeed, lick rates and numbers of trials initiated remained low across Experiment 1B. More work is needed to explain this observation, but prior studies offer some clues. Chronic consumption of high-fat or HF/HS diets has been shown to reduce avidity of rats and mice for sweetened solutions [10,55]. The reduced avidity was associated with lower responsiveness of taste cells to sweeteners [64], independently of any changes in adiposity [55]. Likewise, when rodents chronically consumed a high-fat diet, they experienced an overall reduction in taste cell proliferation and/or taste bud number in both fungiform and circumvallate papillae [56,65,66]. The fact that mice on the control diet regained their high avidity for sweeteners establishes that metabolically deranged mice have the potential to recover their avidity sweeteners. It follows that high daily intake of the sugar solutions (in the experimental diets) must have blocked the recovery process.

Despite exhibiting low avidity for sweeteners during the lick tests, the metabolically deranged mice consumed relatively large quantities of the cellulosic and corn syrups each day. In fact, mass-specific daily intake of these solutions increased across exposure periods 2 and 3. This result corroborates a prior report in which lean B6 mice were exposed to the same experimental diets as used herein [32]. The mice licked progressively less avidly for sweet solutions across the two 28-day exposure periods, but nevertheless consumed progressively greater amounts of the sugar solutions each day.

We can propose two non-mutually exclusive explanations for the absence of a positive relationship between avidity for sweet solutions and daily sugar intake. One is based on the observation that consumption of glucose or sucrose solutions can activate chemosensors in the small intestine. This activation can stimulate appetite and condition flavor preferences in rodents [67,68,69,70]. Accordingly, reductions in orosensory attractiveness of sugar solutions would not necessarily lead to reductions in daily intake of the same sugar solutions. Appetition alone can drive high daily intake. Given that glucose appears to be the main sugar that activates the intestinal chemoreceptors in B6 mice [69,70,71], appetition could also explain why the Gluc-Corn and Gluc-Cell syrups (which contain 99% glucose) stimulated greater daily intake than did the HiFruc-Corn and HiFruc-Cell syrups (which contain 40–45% glucose).

Another explanation for the progressive increase in daily intake of the sugar solutions across exposure periods 2 and 3 is based on the reward-deficiency hypothesis [72,73]. According to this hypothesis, repeated exposure to the sugar solutions could have depressed dopaminergic activity in the striatal reward circuits. If so, then progressively higher intake of the sugar solutions could reflect efforts to compensate for reduced activation of reward circuits.

### 4.5. Strengths and Limitations of Study

The strengths of this study include the fact that we documented metabolic and behavioral impacts of exposure to the HF/HS chow, confirming that it induced metabolic derangement. Second, we used a within-subject design to examine the impacts of switching mice to the experimental or control diets. As a result, each mouse served as its own control. Third, we found that the experimental diets with cellulosic syrups (but not corn syrups) improved insulin function better than did the control diet. In so doing, the experimental diets with corn syrups served as a negative control for the effect of chronically consuming an 11% sugar solution. Fourth, glucose solutions are typically administered orally during GTTs in humans. We used an analogous oral administration method in this study. Because most studies of glucose tolerance in rodents typically administer the glucose challenge intragastrically or intravenously [74,75], they exclude the significant contribution of CPIR to the estimate of glycemic control [41,76]. Fifth, we used a concentration of sugar (11%) that approximates what occurs in many commercial sugar-sweetened beverages. This increased the external validity of our approach.

There are also several limitations of this study. First, exposure periods 2 and 3 spanned 8 weeks. If the exposure period had been longer, then we might have gained deeper insight into the long-term metabolic impacts of the experimental diets. We selected 8 weeks of exposure because it constitutes 7% of the median life expectancy of a laboratory mouse in captivity [77]. Seven percent of the human median life expectancy (=86 years [77]) would be equivalent to an exposure period of ~6 years. Second, the increased consumption of sugar in the modern human diet is derived from both beverages and foods. Our results do not provide insight into the metabolic impacts of sugary foods, which often taste less sweet [38,78]. Third, we could not infer a causal connection between the presence of polyphenols in the cellulosic syrups and improvements in insulin function. For example, additional studies are needed in which metabolically deranged mice are assigned randomly to an 11% Gluc-Corn solution with and without polyphenols. Finally, we studied the B6 strain of mouse. It is possible that our results will not generalize other strains (or species) of rodent or humans, owing to species differences in oral and postoral processing of sugar (e.g., [79]) and capacity to recover from diet-induced obesity (e.g., [80]).

## 5. Conclusions

Despite diet-induced improvements in glycemic control, mice on the experimental diets did not become “healthy.” They either maintained their body weight or became even heavier. By contrast, mice on the control diet lost weight. Further, mice on the Gluc-Cell and HiFruc-Corn diets had higher % body fat (30–31%) than mice on the control diet (~21%) at the end of exposure period 3; and mice on the Gluc-Corn and HiFruc-Cell diets had % body fat values (27–30%) that trended higher than mice on the control diet. Finally, mice on both the experimental and control diets remained hyperinsulinemic throughout Experiment 1B.

For these reasons, we are not proposing that high-sugar diets constitute a viable therapy for recovering from metabolic syndrome. Nevertheless, it is notable that when mice were switched to the experimental diets, they regained glycemic control as quickly as mice on the control diet, despite consuming relatively large quantities of sugar solution each day. Mice on the experimental diets with cellulosic syrups also experienced improvements in insulinemia and insulin sensitivity. More work is needed to identify the mechanisms by which the cellulosic syrups improved insulin function, and whether these mechanisms could be exploited to enhance glycemic control and insulinemia in metabolically deranged humans.

Finally, our results raise important questions about the relationship between avidity for sweet tasting solutions and daily intake of sugar-sweetened foods and beverages. Following exposure to the HF/HS diet, the mice exhibited virtually no attraction to the sweet taste of sucrose or sucralose. Nevertheless, they increased their daily intake of the sugar solutions across exposure periods 2 and 3. It follows that sweet taste was not a critical factor driving the high daily intakes of sugars. The relevance of this observation to humans is supported by a recent report that sweet-taste liking does not reliably predict daily sugar intake in humans [81].

## Figures and Tables

**Table 1:** Carbohydrate composition of the Gluc and HiFruc syrups.

Sugar Syrup	Vendor	Tradename	Constituents	Carbohydrate Profile (% Dry Basis)
Gluc-Corn	Cargill	Clearsweet^®^	Glucose	99
			Fructose	0.1
			Maltose	0.6
			Maltotriose	0.2
			Higher saccharides	0.1
Gluc-Cell	Comet Biorefining	95 DE Dextrose	Glucose	95
			Xylose	~4
			Higher saccharides *	~1
HiFruc-Corn	L&S Sweeteners	55 High- Fructose Corn Syrup	Fructose	>55
			Glucose	>40
			Maltose	<5
HiFruc-Cell	Comet Biorefining	55% Fructose Syrup	Fructose	55 (min)
			Glucose	45 (max)
			Xylose	~4
			Higher Saccharides *	~1

* Oligosaccharides and polysaccharides.

## Data Availability

The data supporting the findings of this study are available from the corresponding author, J.I.G., upon reasonable request.

## Supplementary Materials

The following supporting information can be downloaded at https://www.mdpi.com/article/10.3390/nu17010100/s1; Figure S1. Impact of sex on metabolic and behavioral responses to HF/HS vs. standard chow; Figure S2. Daily intake of the 11% sugar solutions across exposure periods 2 and 3; Table S1. Effect of sex and time on glycemia and insulinemia dynamics; Table S2. Analysis of the effect of sex and sweetener concentration on standardized lick rates for two sweeteners; Table S3. Analysis of the daily intakes of each sugar solution in Figure S2; Table S4. Impact of sex and diet on % adiposity, fat weight, and lean weight; Table S5. Analysis of time-dependent changes in blood glucose and insulin responses across the GTT and exposure periods; Table S6. Analysis of time-dependent changes in relative insulin responses during the postprandial phase of the GTT.
