# Supplementary material for: Fighting Fire with Fire: Impact of Sugary Diets on Metabolically Deranged Mice
Source: Nutrients. 2024 Dec 30;17(1):100. doi: 10.3390/nu17010100 (PMC11722652; doi:10.3390/nu17010100)

## Slide 1
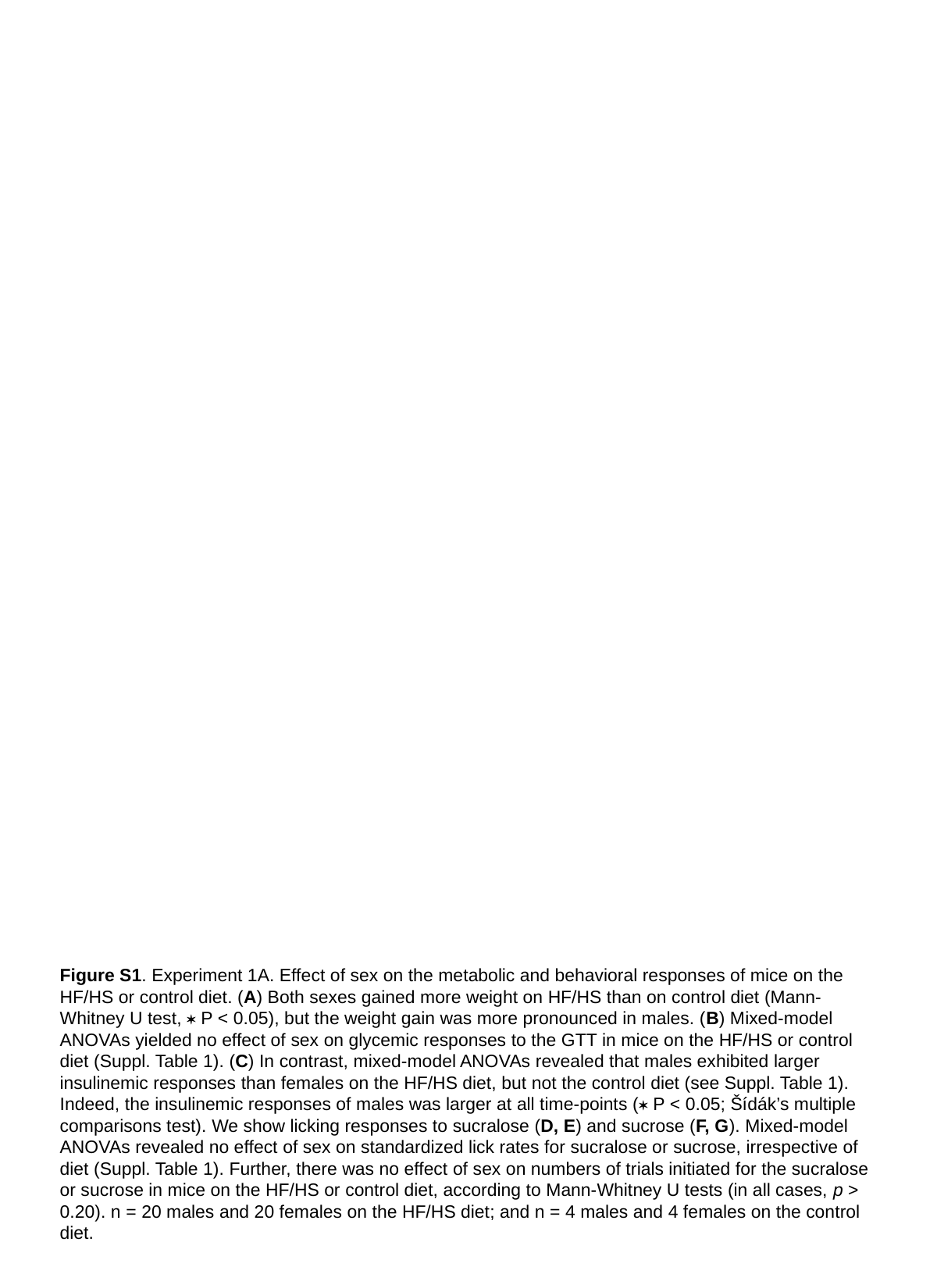

Figure S1. Experiment 1A. Effect of sex on the metabolic and behavioral responses of mice on the HF/HS or control diet. (A) Both sexes gained more weight on HF/HS than on control diet (Mann-Whitney U test, * P < 0.05), but the weight gain was more pronounced in males. (B) Mixed-model ANOVAs yielded no effect of sex on glycemic responses to the GTT in mice on the HF/HS or control diet (Suppl. Table 1). (C) In contrast, mixed-model ANOVAs revealed that males exhibited larger insulinemic responses than females on the HF/HS diet, but not the control diet (see Suppl. Table 1). Indeed, the insulinemic responses of males was larger at all time-points (* P < 0.05; Šídák’s multiple comparisons test). We show licking responses to sucralose (D, E) and sucrose (F, G). Mixed-model ANOVAs revealed no effect of sex on standardized lick rates for sucralose or sucrose, irrespective of diet (Suppl. Table 1). Further, there was no effect of sex on numbers of trials initiated for the sucralose or sucrose in mice on the HF/HS or control diet, according to Mann-Whitney U tests (in all cases, p > 0.20). n = 20 males and 20 females on the HF/HS diet; and n = 4 males and 4 females on the control diet.

## Slide 2
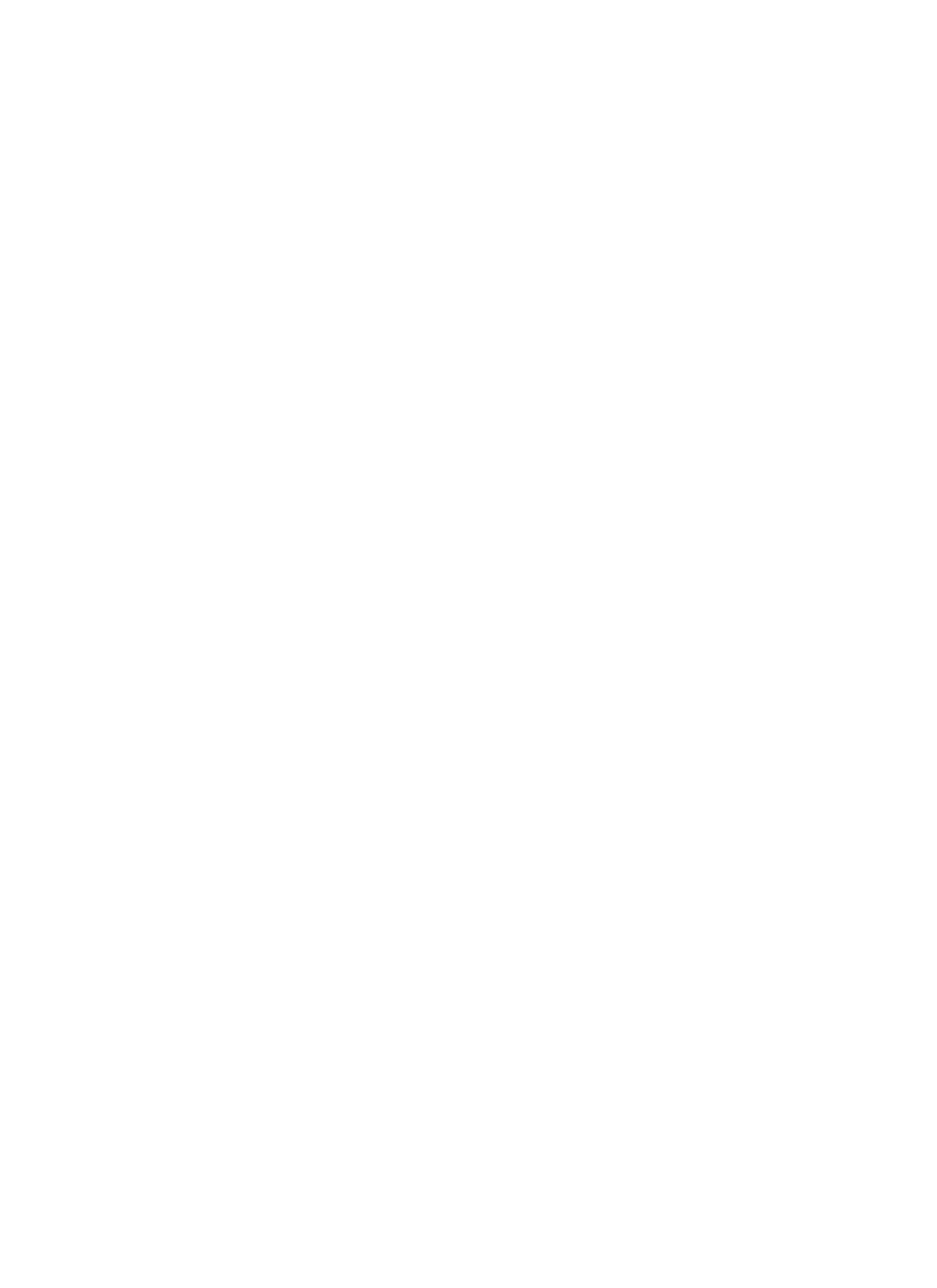

## Slide 3
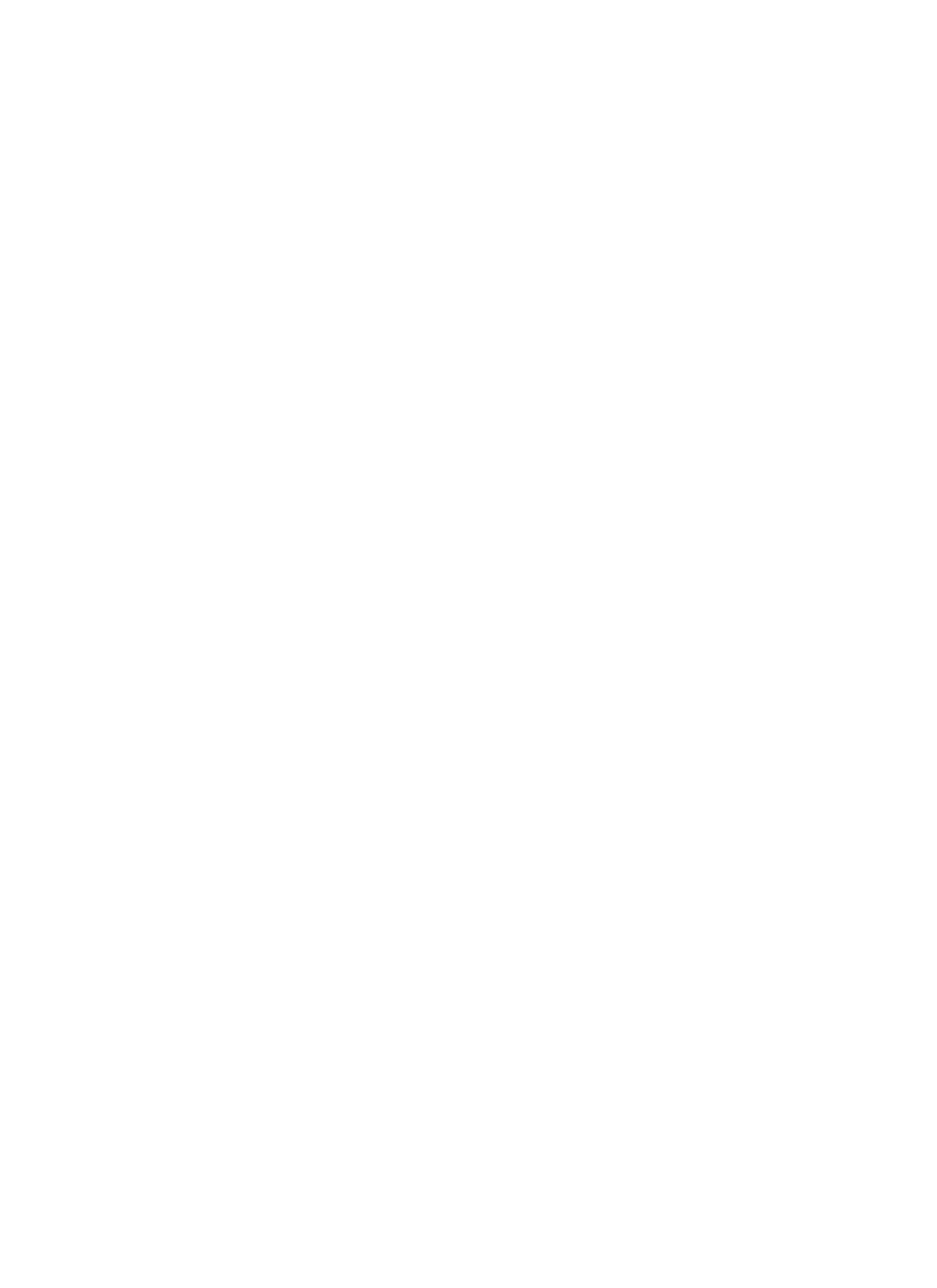

## Slide 4
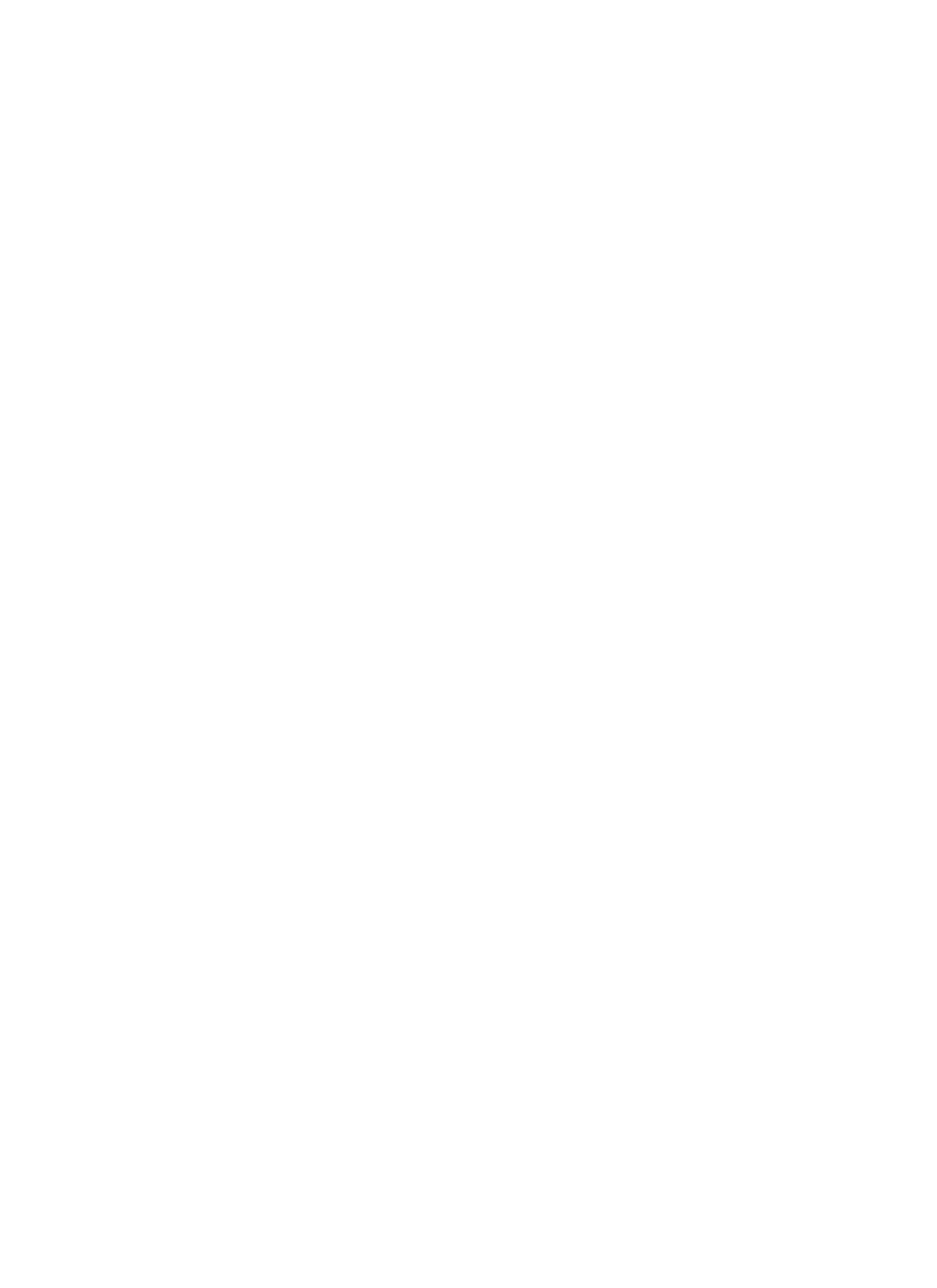

## Slide 5
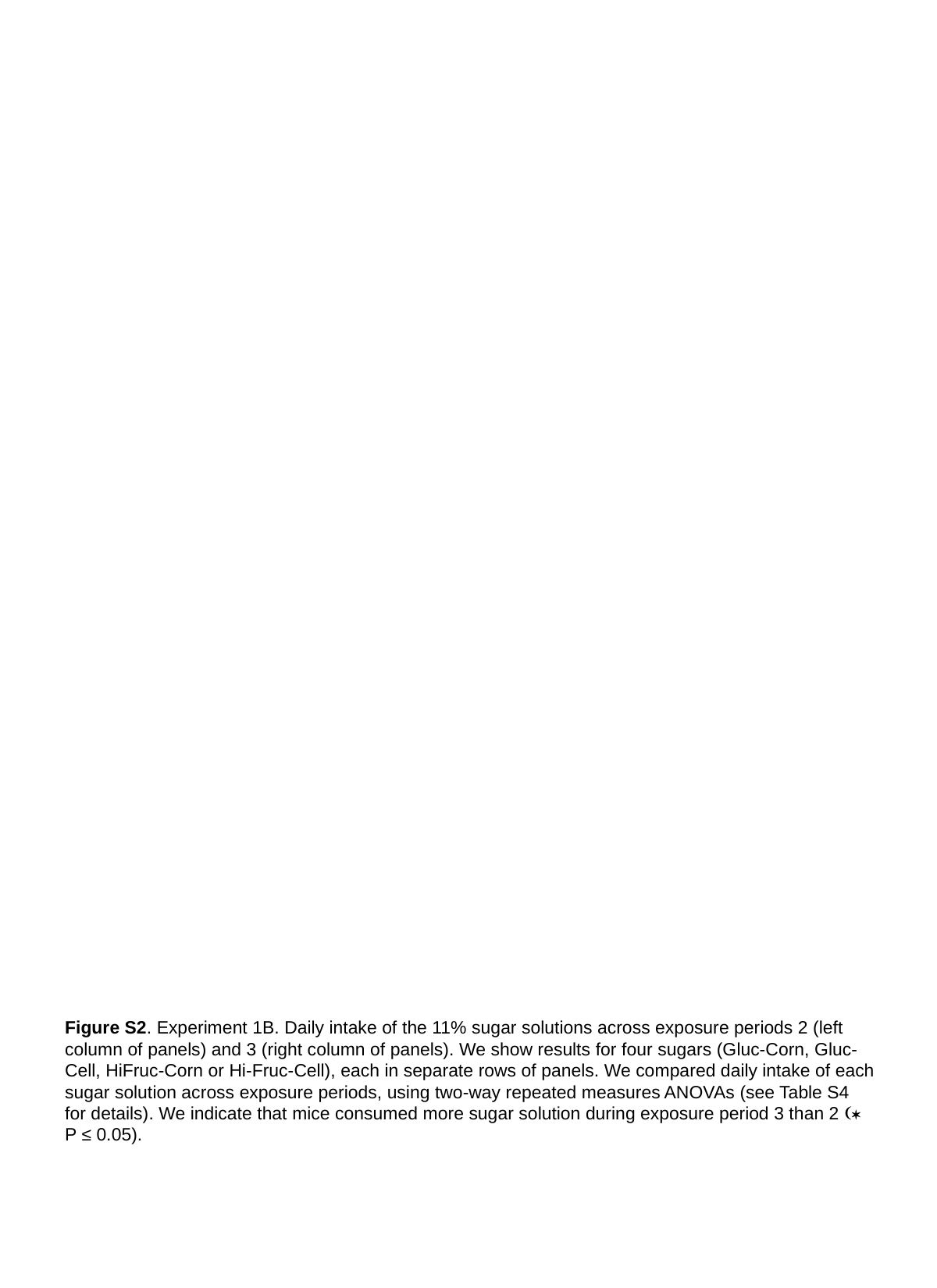

Figure S2. Experiment 1B. Daily intake of the 11% sugar solutions across exposure periods 2 (left column of panels) and 3 (right column of panels). We show results for four sugars (Gluc-Corn, Gluc- Cell, HiFruc-Corn or Hi-Fruc-Cell), each in separate rows of panels. We compared daily intake of each sugar solution across exposure periods, using two-way repeated measures ANOVAs (see Table S4 for details). We indicate that mice consumed more sugar solution during exposure period 3 than 2 (* P ≤ 0.05).

## Slide 6
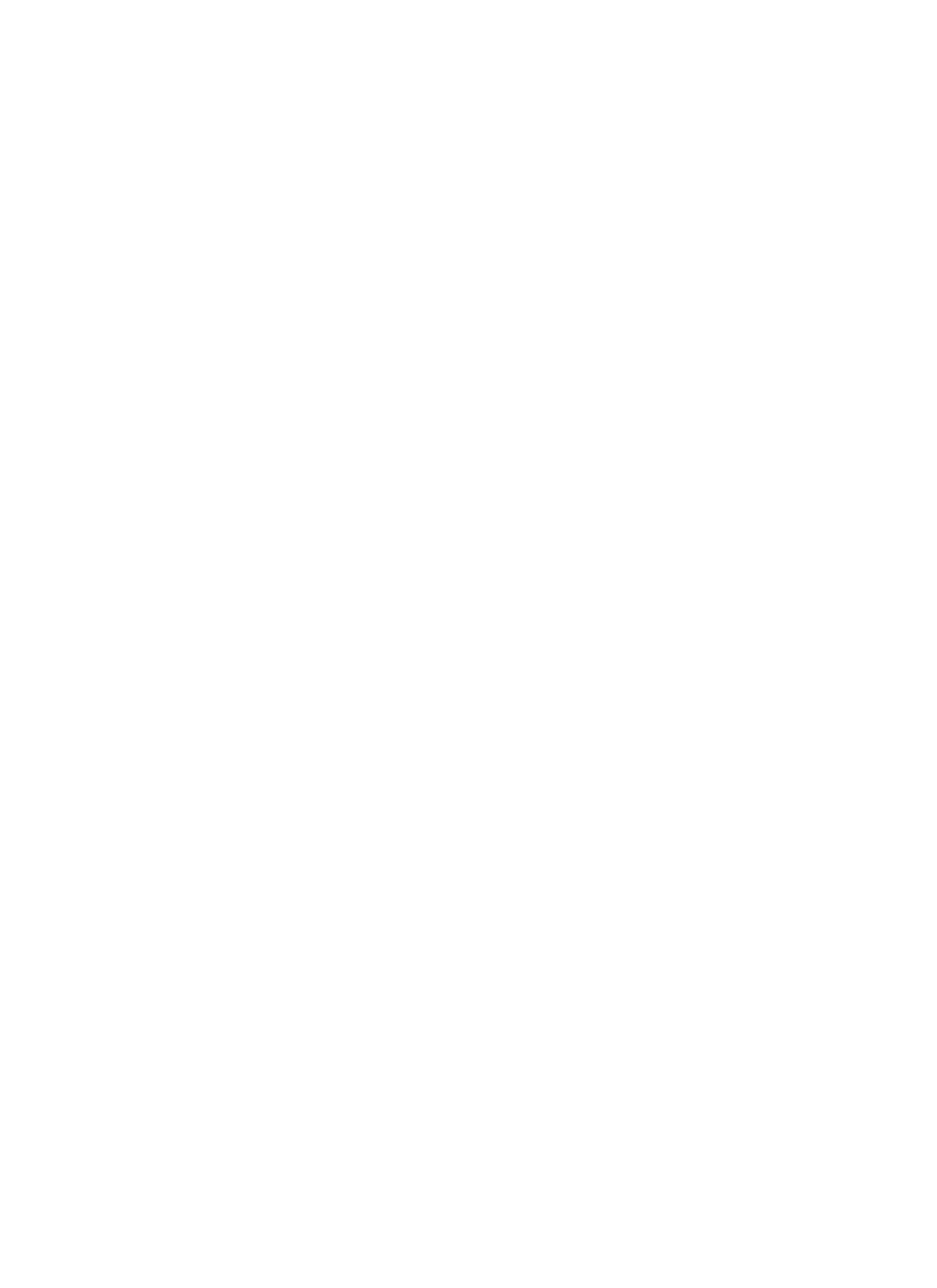

## Slide 7
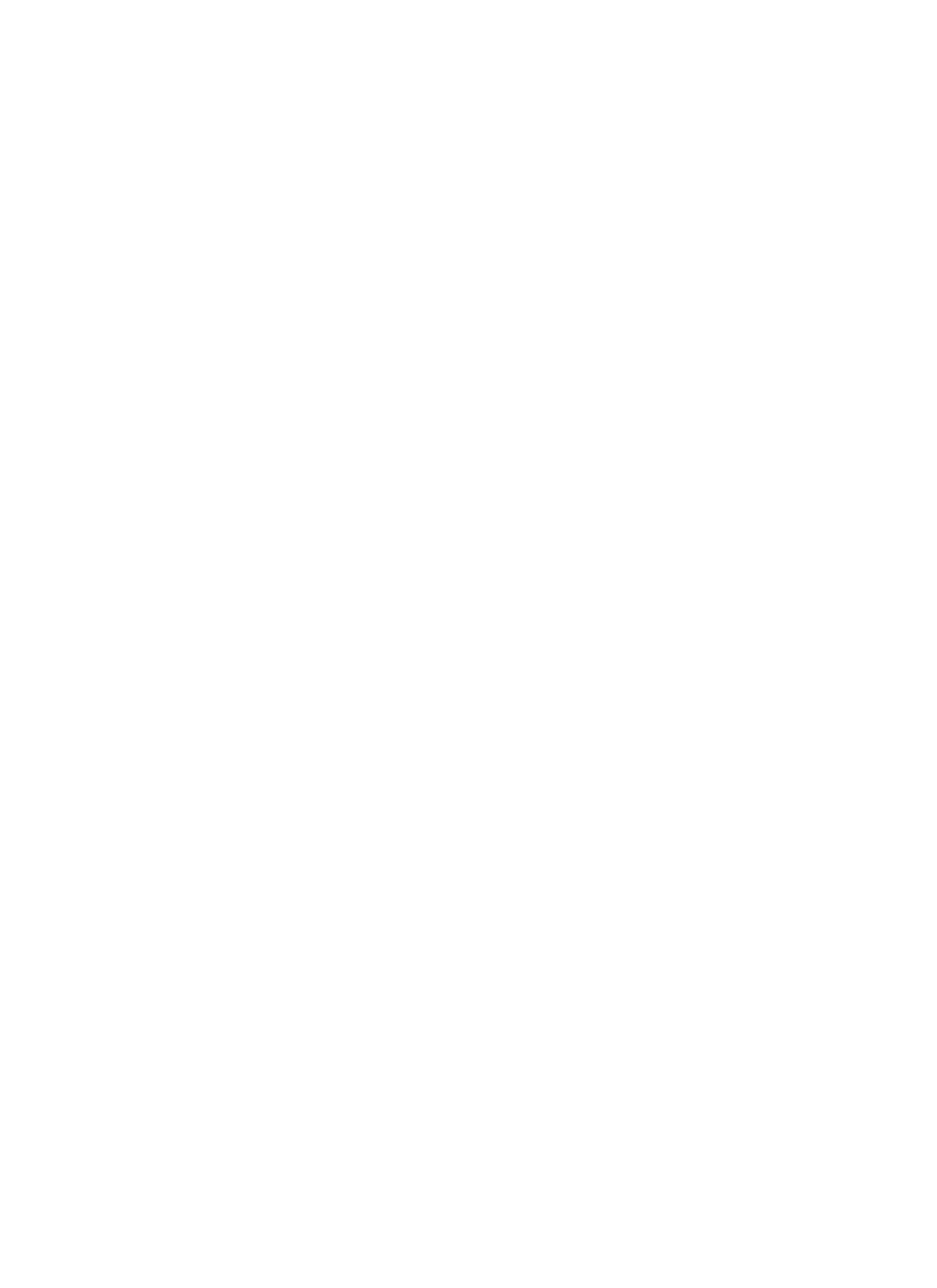

## Slide 8
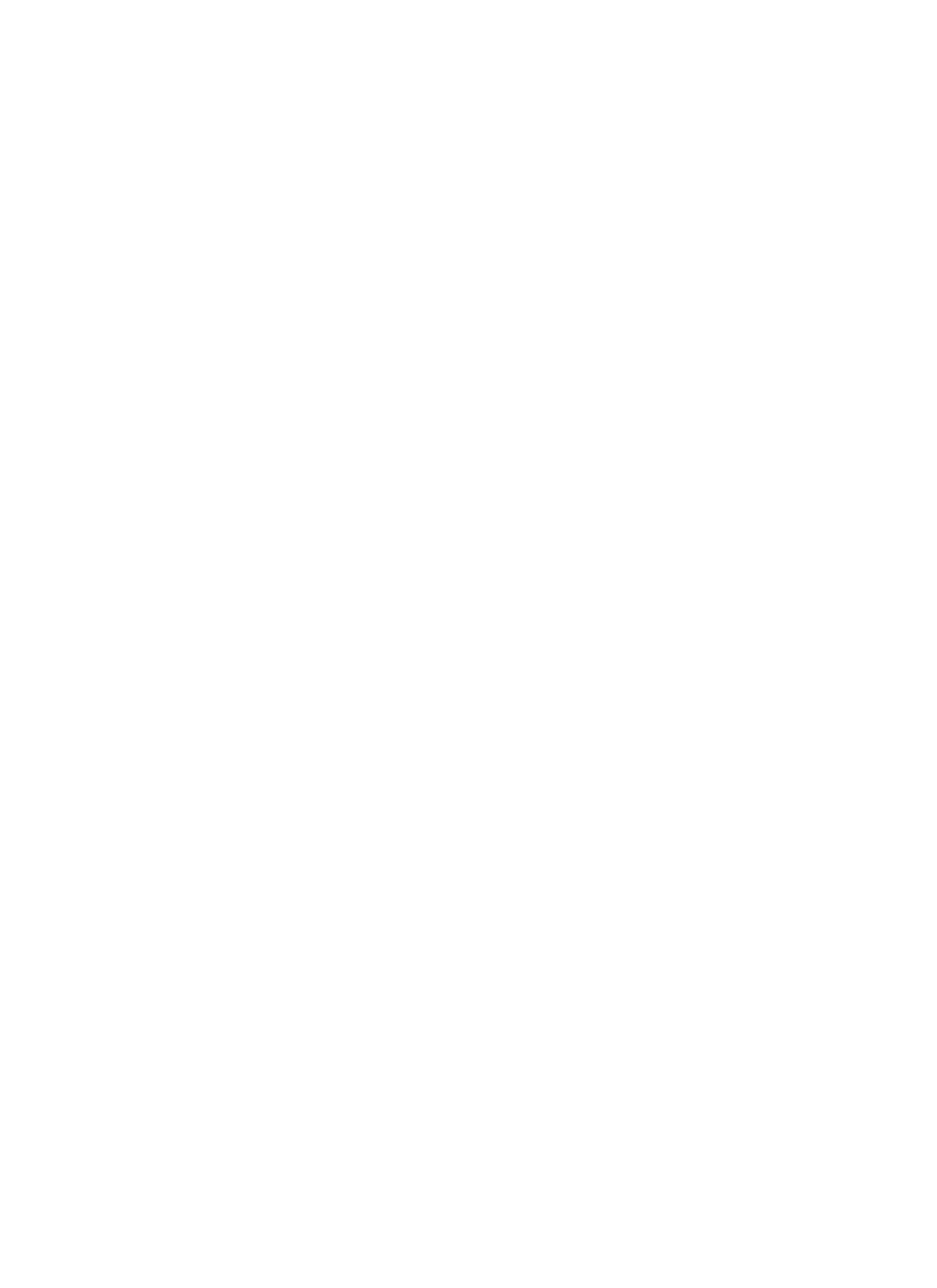

Supplement: Supplementary file 1 [file nutrients-17-00100-s001.zip › nutrients-3346097-supplementary.pptx]
